# Heat shock transcription factor (Hsf) gene family in common bean (*Phaseolus vulgaris*): genome-wide identification, phylogeny, evolutionary expansion and expression analyses at the sprout stage under abiotic stress

**DOI:** 10.1186/s12870-021-03417-4

**Published:** 2022-01-14

**Authors:** Qi Zhang, Jing Geng, Yanli Du, Qiang Zhao, Wenjing Zhang, Qingxi Fang, Zhengong Yin, Jianghui Li, Xiankai Yuan, Yaru Fan, Xin Cheng, Jidao Du

**Affiliations:** 1 College of Agriculture, Heilongjiang BaYi Agricultural University, Daqing, 163319 Heilongjaing China; 2 National Coarse Cereals Engineering Research Center, Daqing, 161139 Heilongjiang China; 3 Crop Resources Institute of Heilongjiang Academy of Agricultural Sciences, Harbin, 150086 Heilongjiang China

**Keywords:** Heat shock transcription factor (Hsf), *Phaseolus vulgaris*, Identification, Sprout stage, Abiotic stress

## Abstract

**Background:**

Common bean (*Phaseolus vulgaris*) is an essential crop with high economic value. The growth of this plant is sensitive to environmental stress. Heat shock factor (Hsf) is a family of antiretroviral transcription factors that regulate plant defense system against biotic and abiotic stress. To date, few studies have identified and bio-analyzed *Hsfs* in common bean.

**Results:**

In this study, 30 Hsf transcription factors (PvHsf1–30) were identified from the PFAM database. The PvHsf1–30 belonged to 14 subfamilies with similar motifs, gene structure and *cis*-acting elements. The Hsf members in *Arabidopsis*, rice (*Oryza sativa*), maize (*Zea mays*) and common bean were classified into 14 subfamilies. Collinearity analysis showed that *PvHsfs* played a role in the regulation of responses to abiotic stress. The expression of *PvHsfs* varied across different tissues. Moreover, quantitative real-time PCR (qRT-PCR) revealed that most *PvHsfs* were differentially expressed under cold, heat, salt and heavy metal stress, indicating that *PvHsfs* might play different functions depending on the type of abiotic stress.

**Conclusions:**

In this study, we identified 30 Hsf transcription factors and determined their location, motifs, gene structure, *cis*-elements, collinearity and expression patterns. It was found that *PvHsfs* regulates responses to abiotic stress in common bean. Thus, this study provides a basis for further analysis of the function of *PvHsfs* in the regulation of abiotic stress in common bean.

**Supplementary Information:**

The online version contains supplementary material available at 10.1186/s12870-021-03417-4.

## Background

Common bean (*Phaseolus vulgaris*) is an essential legume with high economic value [1]. Seeds of common bean are rich in lectin and α-Amylase inhibitors (α-AI) which are used to synthesize pesticides [2] and raw materials for preparation of chemicals [3]. The growth of common bean is sensitive to various abiotic stress stimuli, such as salt, cold and drought [4]. Therefore, it is necessary to improve the tolerance of common bean under abiotic stress.

Heat shock transcription factor (Hsf) regulates signal transductions associated with heat stress in plants [5]. Hsfs participate in signal reception and transmission, regulation of gene expression, resistance to stress and heat tolerance in plants. Members of the Hsf family have five conserved domains [6]: DNA-binding Domain (DBD), oligomerization domain, (OD), C-terminal activation domain (CTAD), nuclear localization signals (NLS), and nuclear export signals (NES) [7]. Among them, DBD and OD domain are the most evolutionary conserved and are used to accurately identify and combine the promoters of heat shock protein (Hsp) [8]. The C-terminal activation domain contains a repressor domain: LFGV-peptide [9], which directly regulates the expression of genes in response to heat shock. The NLS and NES modulate the transnuclear transport of Hsf protein and free distribution of proteins in the nucleus and cytoplasm. Hsf can activate the heat shock protein (Hsp) thereby promote refolding, assembly, distribution and degradation of damaged proteins. In this way, it helps plants to resist abiotic stress [10].

The first *Hsf* gene was cloned in yeast. Since then, several mammalian *Hsf* genes have been identified. In plants, the first *Hsf* gene was cloned in tomato (*Solanum lycopersicum*) [6]. To date, *Hsfs* have been reported in several species, including *Arabidopsis* [11], rice (*Oryza sativa*) [12], soybean (*Glycine max*) [13], maize (*Zea mays*) [14] due to the continuous improvement of genome sequencing technology. The number of *Hsf* genes varies across plants. For instance, wheat, soybean, maize and *Arabidopsi*s have 56, 52, 30 and 21 genes, respectively [7].

*Hsf* gene members are involved in the regulation of growth and development in plants. Carotenoids and chlorophyll have been shown to promote plant photosynthesis in transgenic tobacco by upregulating *HsfA9* expression [15]. A total of 14 *Hsfs* have been identified in *Citrus*, all of which participate in fruit development and maturation. For instance, *CrHsfB2a* and *CrHsfB5* were found to regulate citrate content [16]. Various stimuli of biotic and abiotic stress can upregulate expression level of *Hsfs*, hence influence plant response to stress. The expression of *HsfA2*, *HsfB1*, *HsfA4a*, *HsfB2a*, *HsfB2b* and *HsfA7a* in *Arabidopsis* has been shown to increase with temperature. However, high temperatures do not increase expression of some *Hsfs* [11]. Previously, 19 *Hsf* members*,* including *VHsf01*, *VHsf05*, *VHsf15* and *VHsf18* were found to exhibit different gene expression patterns between resistant and susceptible grape species under high-temperature stress (47 °C) and heat adaptation (38 °C). The differential expression of these genes may explain differences in heat tolerance among various grape species [17]. Different types of biotic and abiotic stresses including cold, drought, pests and diseases increase the expression of *Hsf* members*,* such as *HsfB3a*, *HsfA6a*, *HsfA2a* and *HsfA9b* in Cassava (*Manihot esculenta Crantz*) [18]. It has also been reported that salinity, osmotic pressure and cold stress increase the expression of *HSFA6b*, an ABA-mediated stress response gene in *Arabidopsis*, [19]. Heat, drought and salt stress similarly alters the expression level of *Hsf* genes in *Populus euphratica* [20].

In the study, 30 members of *Hsf* gene family were identified in common bean. These members’ phylogeny, motif, gene structure, evolutionary expansion and expression patterns of the identified genes were analyzed at the sprout stage. Our bionformatic analysis provide insights into *PvHsfs* and give useful information for further functional dissection of *PvHsfs* in common bean.

## Results

### Identification of Hsf members in *P. vulgaris*

**Fig. 1:**
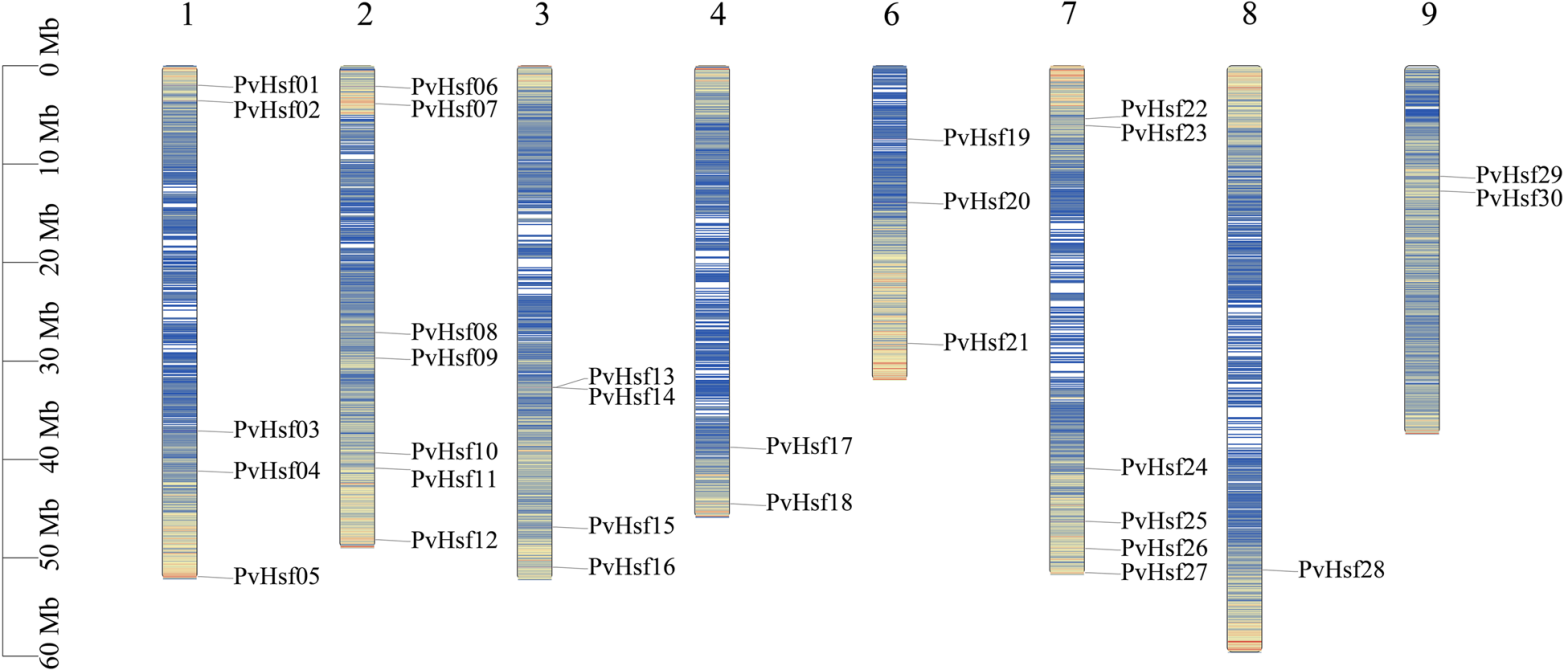
Chromosomal location of PvHsf members. The nine ribbons represent nine chromosomes containing the PvHsf members. The blue lines show the number of genes

The hmm search identified 35 protein sequences in the reference genome of *P. vulgaris.* Among them, 30 were found to be members of the Hsf family after removal of duplications. The 30 protein sequences were located on nine chromosomes of *P. vulgaris* except LG10 and LG11. The Hsf members were named based on their chromosomal location (e.g., PvHsf1-PvHsf30) (Fig. 1). PvHSF06 had the longest protein length (490) and CDS length (1470) whereas PvHSF12 had the shortest protein length (206) and CDS length (618). The isoelectric point of PvHsf members ranged from 4.82 to 9.08, and the molecular weight ranged from 23,907.29 to 54,538.32. The detailed information of PvHsf members is shown in Table S1.

### Evolutionary analysis of PvHsfs

**Fig. 2:**
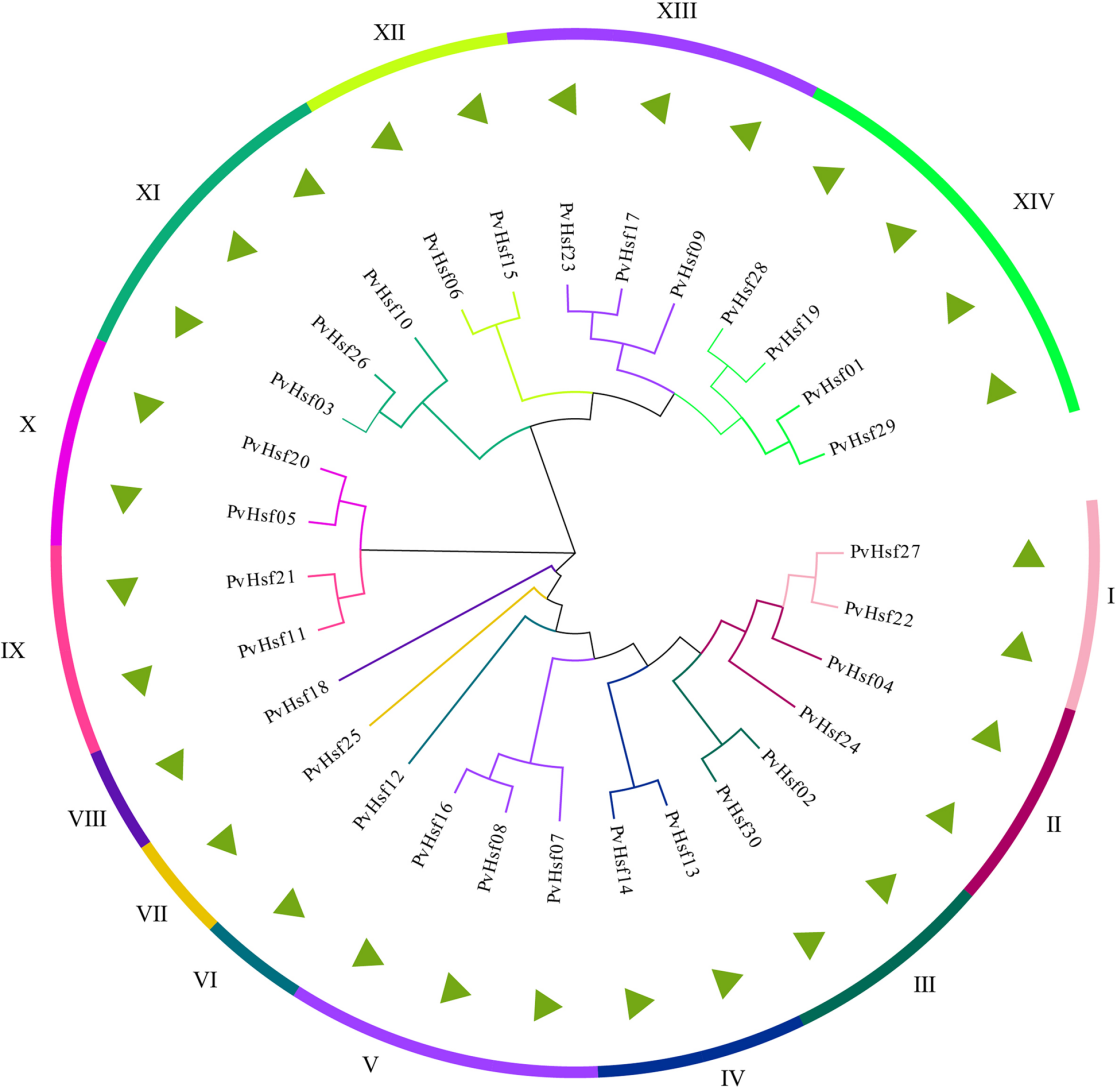
Evolutionary analysis of PvHsf members. Different colors show different subgroups

MEGAX software was used to analyze the protein sequence of PvHsf members using the Maximum Likelihoodphy function and jtt + g + i model predicted by MEGA. The 14 subgroups were divided based on the analysis result of PvHsf members (Fig. 2). In these 14 subgroups, the subgroup VI, VII and VIII had only one PvHsf member. The subgroup XIV had the highest number of PvHsf members (4).

### Motifs and gene structure of PvHsfs

**Fig. 3:**
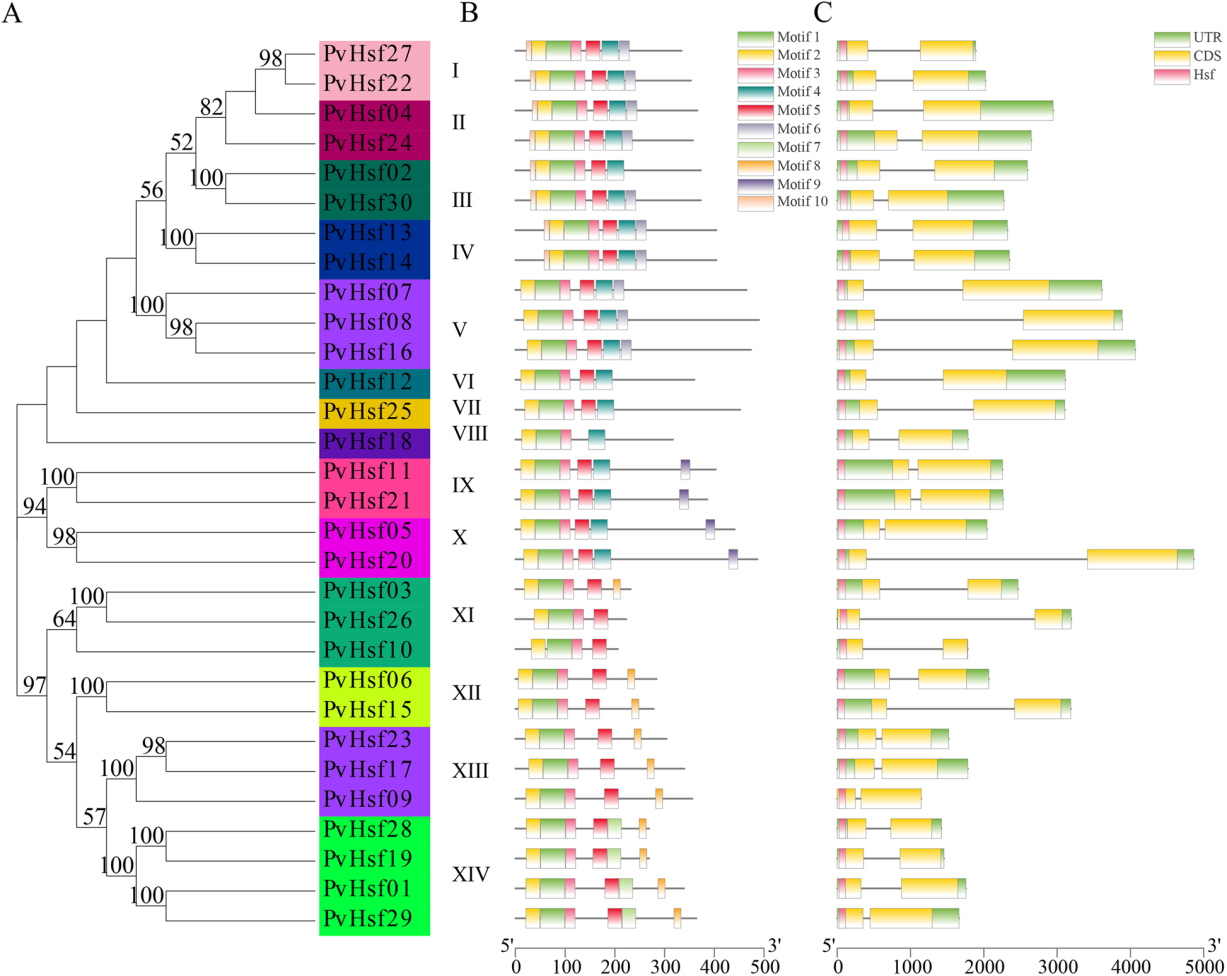
The motifs and gene structure of PvHsf members. (A) Evolutionary relationships among PvHsfs. Different colored shades represent different subgroups (I-XIV). (B) The 10 motifs are represented by different colors. (C) The gene structure of *PvHsfs*. The pink, yellow and green squares represent Hsf structure, CDS and UTR respectively. The black line represents intron

Using MEME tool, 10 motifs of PvHsf members were identified (indicated using squares with different colors) and the sequence of each motif is shown in Fig. S1. The location of squares represents the location of each motif (Fig. 3A and B). Motifs- 1, 2, 3 and 5 were identified in all subgroups of PvHsf. Subgroup I, II, III and IV had similar motifs which were found in different locations. Motif-10 was not found in subgroup V while motif-9 was only found in subgroup IX and X. Motif-7 was found in subgroup XIV whereas motif-8 was present in subgroup XI, XII, XIII and XIV. Each subgroup PvHsf members had similar motifs. The structures of exons and introns of *PvHsfs* were determined using the GSDS (Fig. 3C). Each subgroup members had a conserved Hsf domain (pink box), and the location of exon and intron were similar in each subgroup.

### Evolutionary analysis of four species

**Fig. 4:**
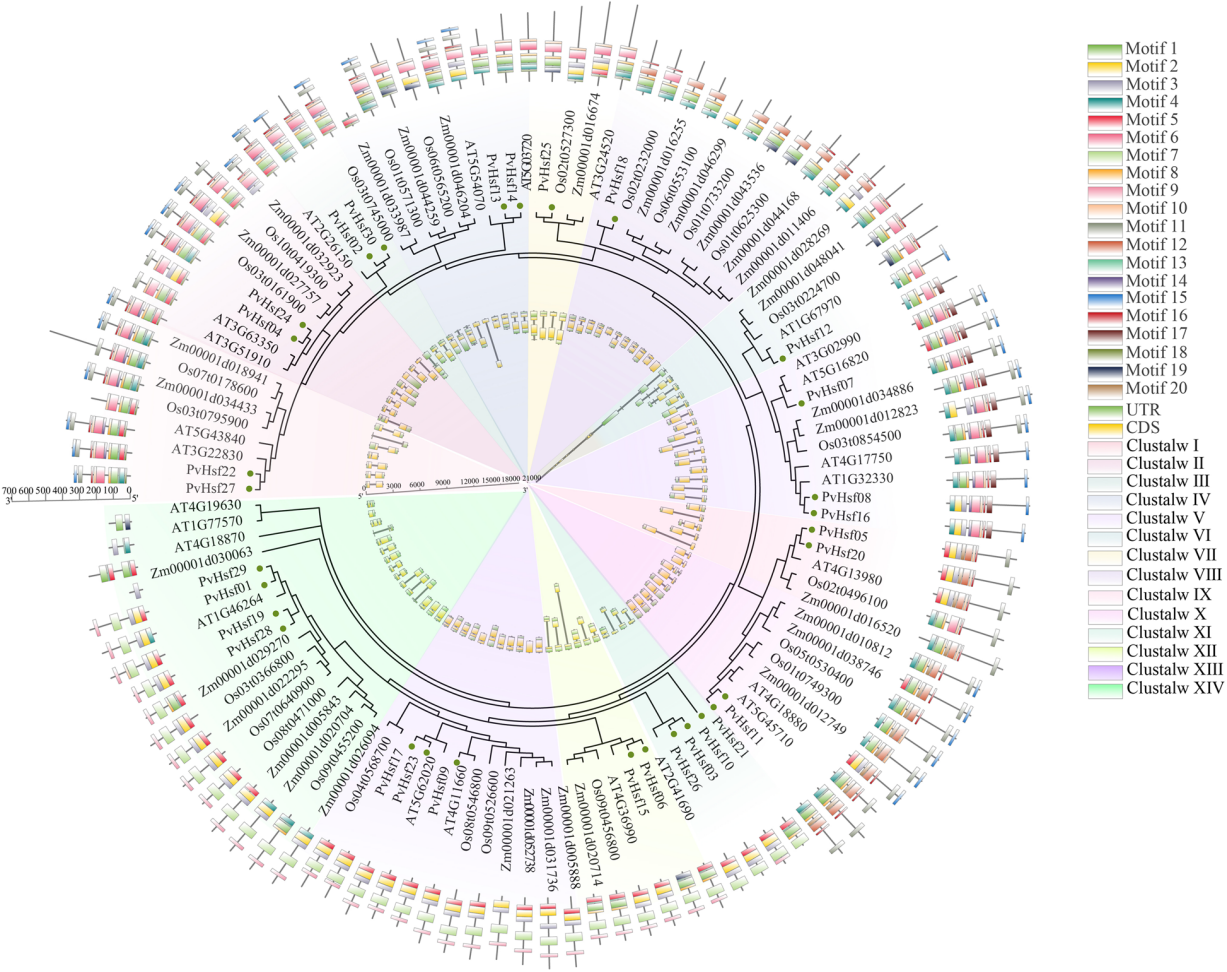
Evolutionary analysis of Hsf members of four species (*P. vulgaris*, *O. sativa*, *Z. mays* and *Arabidopsis*). Fourteen different colored shadows represent different subgroups. The outer ring indicates the motifs of Hsf members and the inner ring shows the gene structure of Hsf members. Black circles show the PvHsf members

In this study, members of PvHsf genes were identified in *P. vulgaris* whereas members of Hsf genes in plants, including monocotyledonous (*O. sativa* and *Z. mays*) and dicotyledonous (*Arabidopsis*) were retrieved from published research [11, 12, 14]. Also, the jtt + g + i model was the best model to find the evolutionary relationship between Hsf members in four species, which predicted by MEGAX. The four species Hsf members were also devided into 14 subgroups while subgroup III and XI had not monocot members; 20 motifs were identified from the four species Hsf members in motif analysis (Fig. S2), although the order of motifs had changed compared with PvHsfs, the structure of motifs was similar. Motif-1, 2 and 3 encoded DBD domain while motif-9 encoded -LFGV- motif (C-terminal activation domain); Most Hsf members also had two exons, which the results of gene structure was similar with PvHsfs; The results of evolution, gene structure and motif analyses revealed that members of Hsf in each subgroup had similar motifs and gene structure (Fig. 4).

### Cis-elements analysis of PvHsfs

**Fig. 5:**
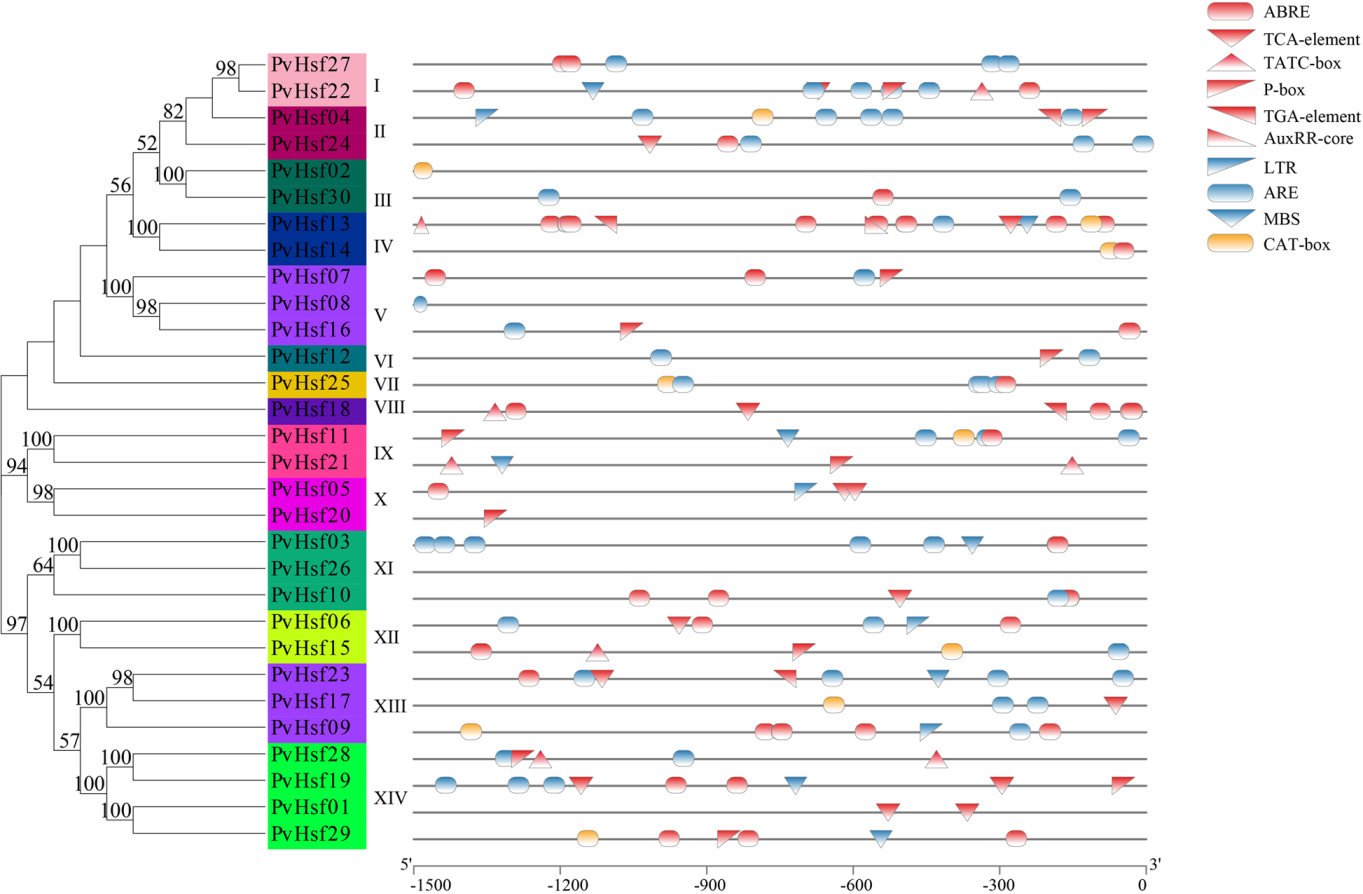
The *cis*-elements of *PvHsfs.* **A** The evolutionary relationships among *PvHsfs*. The different colored shades represent different subgroups. **B** The *cis*-elements of *PvHsfs.* The red models represent elements associated with hormone responsiveness. The blue models represent elements associated with stress-inducibility. The orange models represent elements involved in germination

Using plantCARE, 10 *cis-*elements were identified in *PvHsfs*. These *cis-*elements found to be involved in the regulation of hormone responsiveness, environmental stress and germination (Table S2). Elements marked in red such as ARBE, TCA-element, TATC-box, P-box, TGA-element and AuxRR-core were hormone-related elements; Three elements marked in blue (LTR, ARE, MBS) were stress-related elements whereas CAT-box was the germination-related element. These results showed that *PvHsfs* might regulate hormone responsiveness, environmental stress and germination (Fig. 5).

### Collinearity and Ka/Ks

**Fig. 6:**
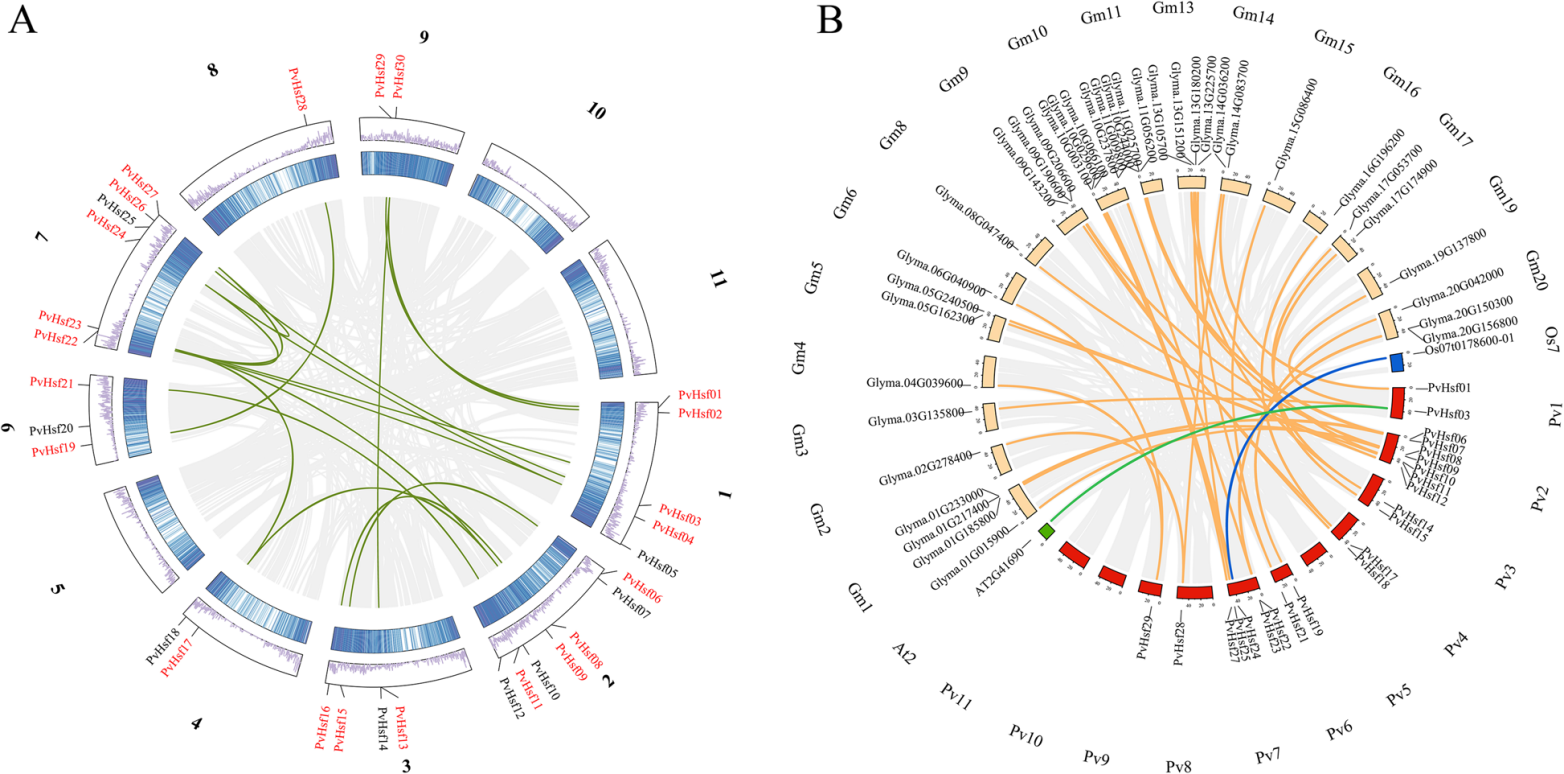
Collinearity analysis of *PvHsfs.* **A** Collinearity among *PvHsf* members in common bean (*P. vulgaris*). The *PvHsfs* marked in red had collinearity while those marked in black lacked collinearity. The middle two rings represent the gene density of each chromosome. Gray background line represents collinear background. Green lines represent collinear relationships among *PvHsf* members. **B** Collinearity of *PvHsf* members in soybean, rice and *Arabidopsis*. Red, orange, blue and green boxes represent the LGs of *common bean*, soybean, rice and *Arabidopsis* respectively. Gray lines indicate the total collinearity background. Orange, blue and green lines show the collinearity of *PvHsfs* with genes of soybean, rice and *Arabidopsis*

*PvHsfs* had 16 pairs of collinearity. *PvHsf22* and *PvHsf27* exhibited the most pairs (3 pairs) (Fig. 6A). Compared to *Arabidopsis* (1) and rice (1), *PvHsfs* in soybeans had more collinear genes (36), indicating a close association between the common bean and soybean (Fig. 6B). KA/KS analysis revealed that there were no two pairs of *PvHsfs* (Table S3), indicating that *PvHsfs* eliminated harmful mutations, while maintaining the protein (purity selection).

### In Silico tissue-specific expression analysis

**Fig. 7:**
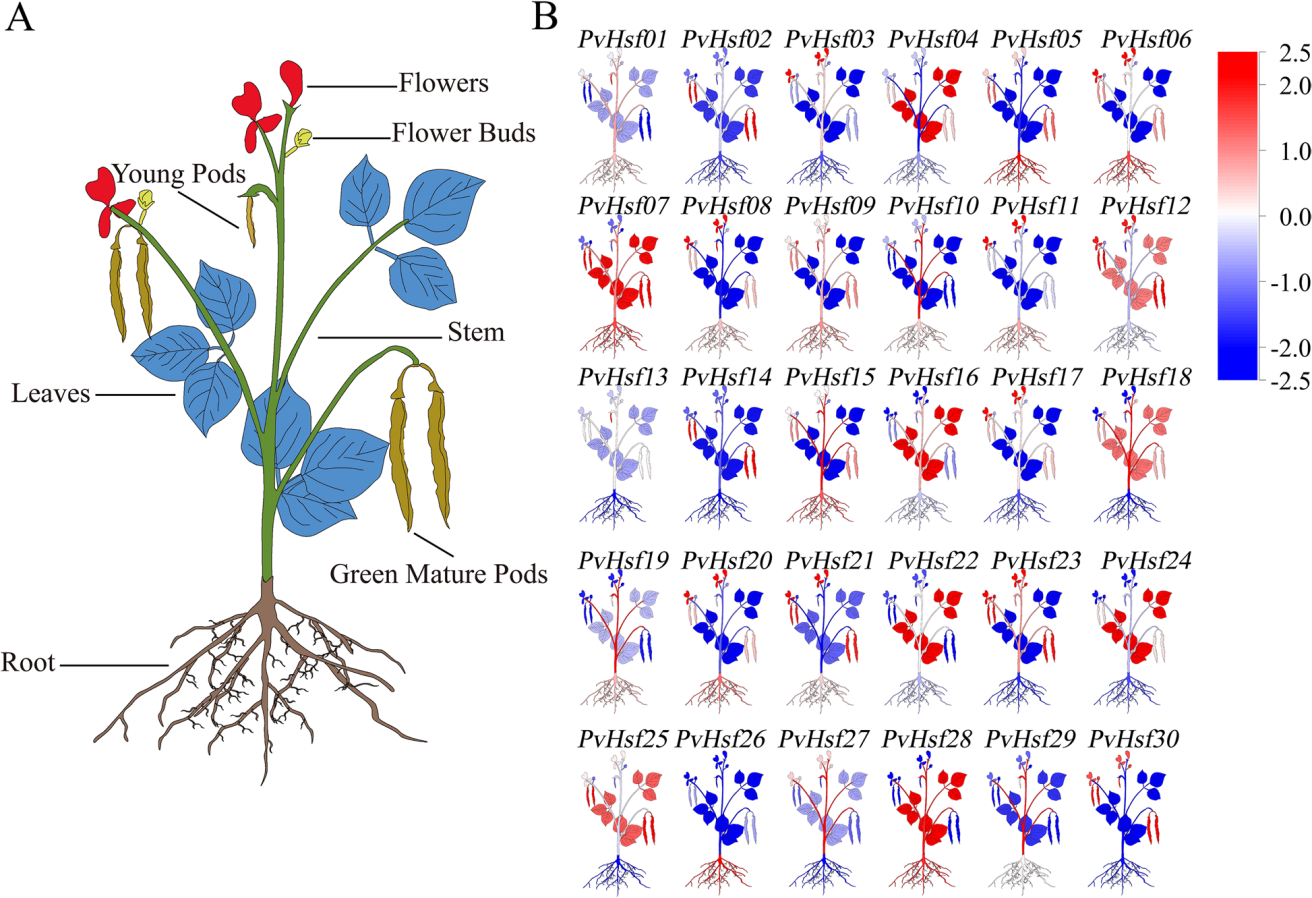
The tissue-specific expression analysis of *PvHsfs*. **A** Schematic illustration of different tissues of common bean (*P. vulgaris*). **B** A cluster map of each *PvHsf*. Red indicates high gene expression level of *PvHsfs* while blue shows low gene expression level of *PvHsfs*

Tissue-specific expressions of *PvHsfs* were obtained from the phytozome database. The expression levels of *PvHsfs* in flowers, flower buds, young pods, leaves, green mature pods, stems and roots were as shown in Fig. 7. All *PvHsfs* members were specifically expressed in different tissues, with various *PvHsfs* members exhibiting marked variations in expression in different tissues. For instance, compared to other tissues, *PvHsf02* was highly expressed in green mature pods; Compared with other different tissues, *PvHsf05* was highly expressed in roots and pods higher than other tissues; *PvHsf03* was highly expressed in flowers, stems and young pods; *PvHsf07* levels were suppressed in flowers, flower buds and young pods while *PvHsf28* levels were elevated in leaves, stems and roots*.*

### Tissue-specific expression analysis at the sprout stage

**Fig. 8:**
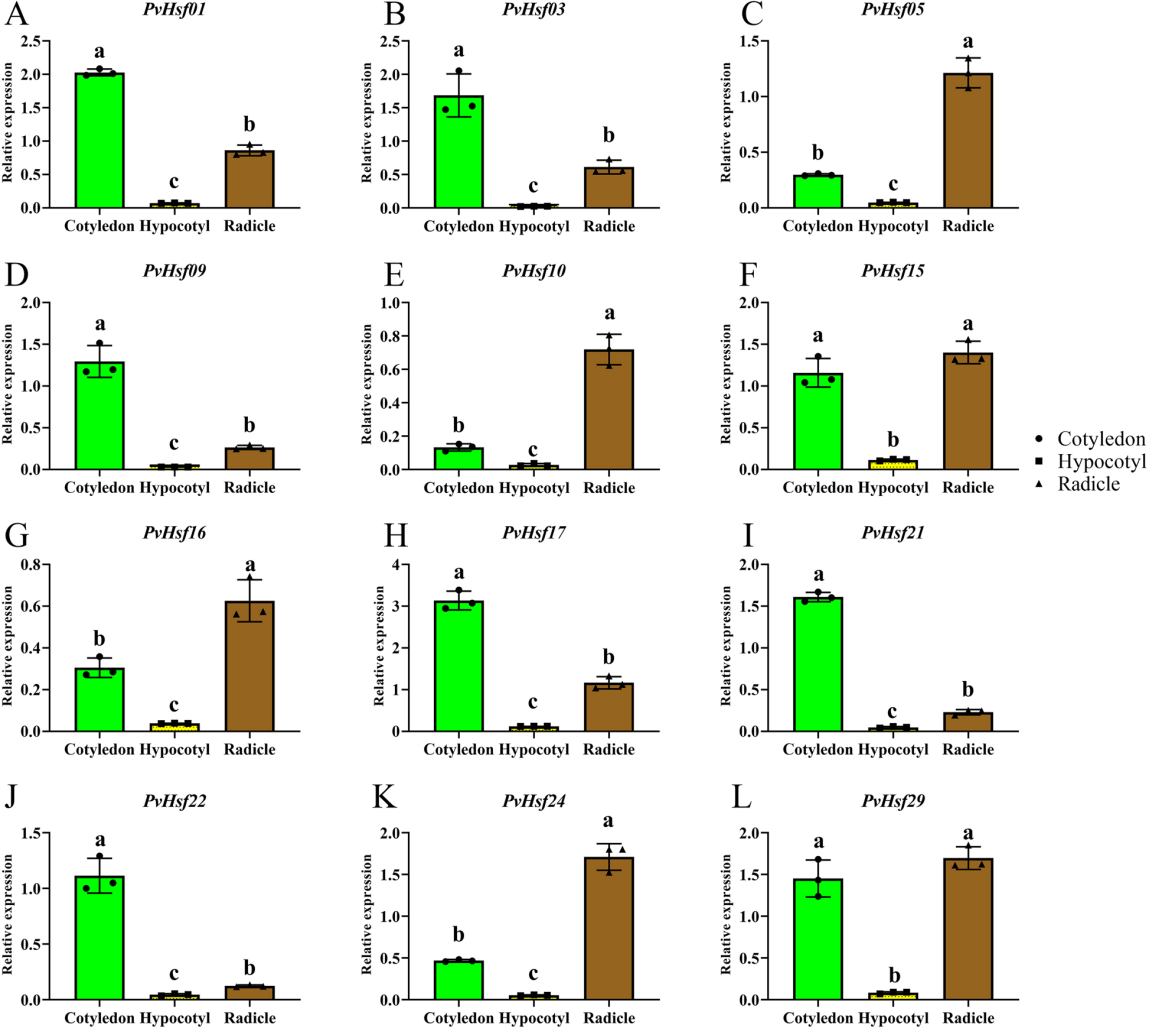
The expression of *PvHsfs* in cotyledon, hypocotyl and radicle at the sprout stage. Green, yellow and brown squares indicate the relative mRNA expression of *PvHsfs* in cotyledon, hypocotyl and radicle of common bean (*P. vulgaris*)

The expression of *PvHsfs* in cotyledon, hypocotyl and radicle at the sprout stage was also analyzed. The expression levels of *PvHsfs* were specific in the cotyledon, hypocotyl and radicle. At the sprouting stage, expression levels were relatively low in the hypocotyl, relative to cotyledons and radicles (Fig. 8). Some *PvHsfs*, including *PvHsf01*, *PvHsf03*, *PvHsf09*, *PvHsf17*, *PvHsf21* and *PvHsf22* were highly expressed in the cotyledons while some *PvHsfs,* including *PvHsf05*, *PvHsf10*, *PvHsf16* and *PvHsf24* were highly expressed in both cotyledons and radicles. These results imply that cotyledons and radicles are potential target tissues for studies on *PvHsfs* at the sprouting stage.

### Stress-associated expression levels

**Fig. 9:**
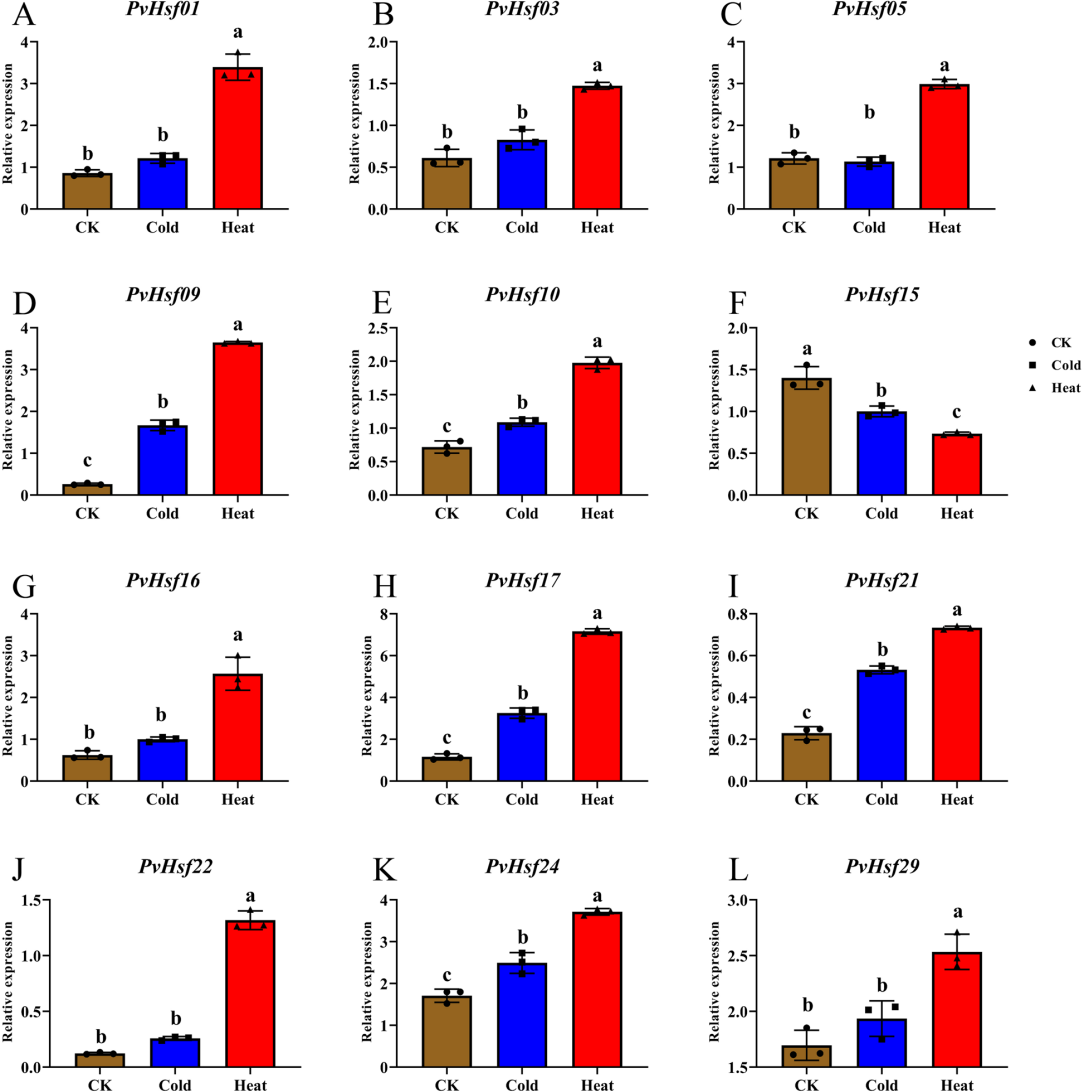
The expression of *PvHsfs* in radicle of common bean (*P. vulgaris*) at the sprout stage under cold stress (4 °C) and heat stress (45 °C). The brown, blue and red squares indicate the relative mRNA expression of *PvHsfs* in radicle under cold stress and heat stress

QRT-PCR was used to assess the expressions of *PvHsfs* under heat and cold stress conditions. Stress dysregulated the expression levels of *PvHsfs*, with specific characteristic expressions under different stressors. Under heat stress, *PvHsfs* levels, apart from *PvHsf15* levels, were markedly elevated under heat stress except *PvHsf15* (Fig. 9). In CK and under cold stress conditions, there were no significant changes in the expressions of dome *PvHsfs,* such as *PvHsf01*, *PvHsf03*, *PvHsf05*, *PvHsf16* and *PvHsf29.* However, these levels were significantly altered under heat stress, indicating that these *PvHsfs* are highly responsive to heat stress, when compared to cold stress.

**Fig. 10:**
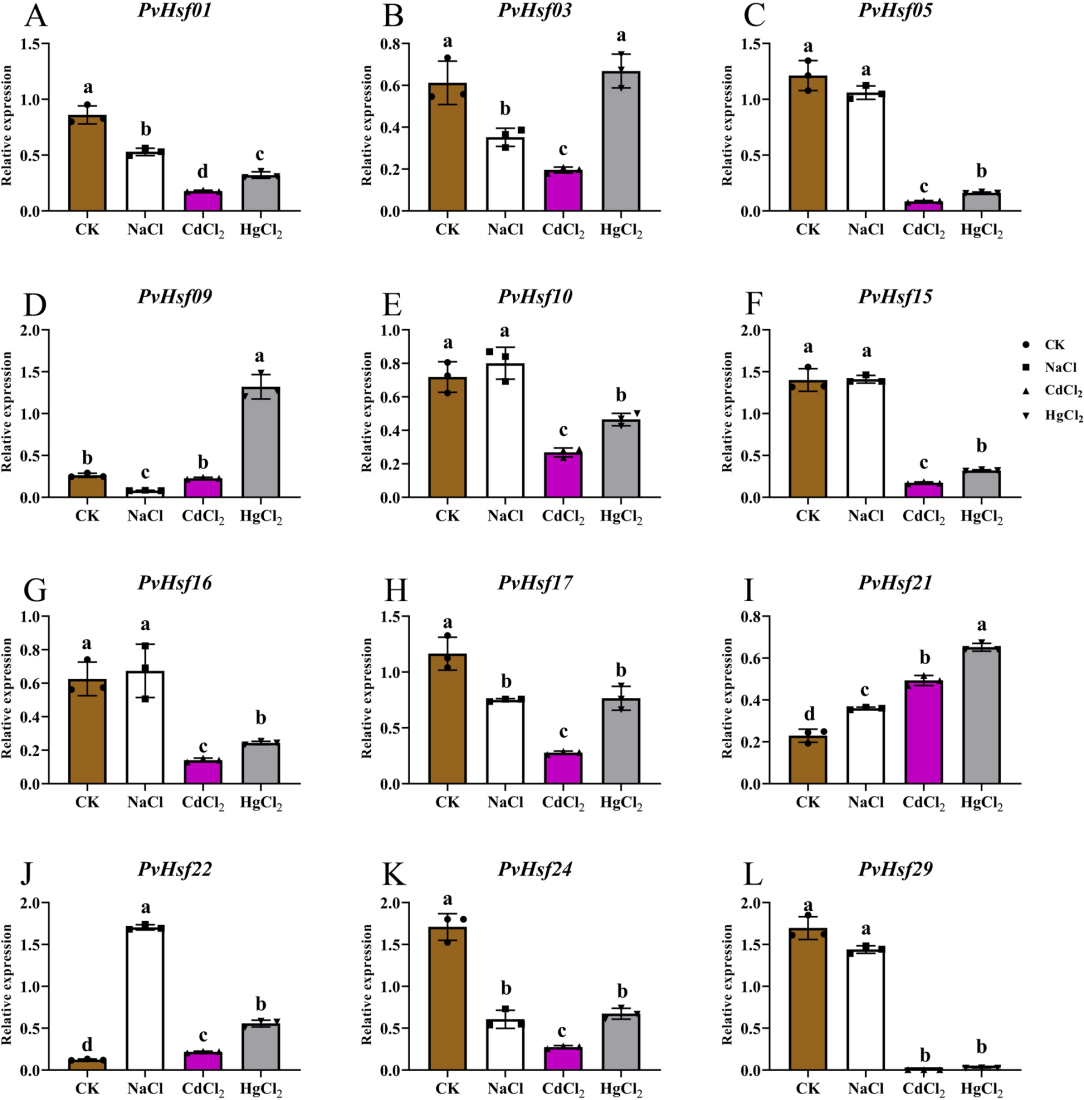
The expression of *PvHsfs* in radicle of common bean (*P. vulgaris*) at the sprout stage under salt stress (NaCl) and heavy metal stress (CdCl_2_ and HgCl_2_). Brown, white, purple and gray squares indicate the relative mRNA expression of *PvHsfs* in radicle under salt, CdCl_2_ and HgCl_2_ stress

QRT-PCR was also performed to evaluate the expressions of *PvHsfs* under salt and heavy metal stressors (Fig. 10). Under salt stress conditions, *PvHsf21* and *PvHsf22* levels were significantly elevated, however, *PvHsf01*, *PvHsf03*, *PvHsf09*, *PvHsf17* and *PvHsf24* levels were markedly suppressed. Moreover, exposure to Cd^2+^ stress suppressed the levels of most *PvHsfs,* apart from *PvHsf09*, *PvHsf21* and *PvHsf22*. Under salt and heavy metal stressors, levels of most *PvHsfs* were significantly dysregulated, apart from those of *PvHsf03. PvHsf01, PvHsf17, PvHsf21, PvHsf22* and *PvHsf24*. Therefore, *PvHsfs* can be used as candidate members of the Hsf family for studies at sprouting stages under stress.

## Discussion

Various species have different Hsf members. There are 29 Hsf members in Tartary buckwheat (*Fagopyrum tataricum*) [21], 28 members in poplar (*Populus*) [22], 21 members in *Arabidopsis* [23], 19 members in grapes (*Vitis vinifera*) [24], 18 members in tomatoes (*Solanum lycopersicum*) [7] and 16 members in alfalfa (*Medicago sativa*) [22]. Duplication of gene family members during plant evolution is associated with genomic replication events [17]. In this study, 30 Hsf family members were identified in the genome of the common bean (PvHsf1-PvHsf30) (Fig. 1). Compared to other dicotyledons, the common bean was established to have more Hsf members. These PvHsfs were all found to be located in 11 linkage groups. Therefore, based on these results, after differentiation from early ancestors, the common bean may have had more genomic replication events.

PvHsfs in each subgroup exhibited similar motifs. Motifs-1, 2 and 3, encoding the DBD domain, were present in all PvHsfs subgroups. This domain was the most conserved and had a three helical-bundle structure [25]. Subgroup X and XI exhibited motif-9 (AHA domain) [23] while motif-8, a characteristic C-terminal motif (−LFGV-), which functions as a repressor domain, was found in PvHsf members of subgroup XI, XII, XIII and XIV [9]. Comparable findings, whereby Hsf members in each subgroup had similar motifs, were found in *Populus trichocarpa* [26], cotton (*Gossypium hirsutum*) [27] and peach (*Prunus persica*) [28].

Introns regulate gene expressions [29]. Therefore, it is important to elucidate on gene functions by analyzing gene structures [30]. Analyses of gene structures of *PvHsf* members revealed that all *PvHsfs* had more than one intron. However, intron lengths were different for each subgroup, and each subgroup member exhibited a similar structure. The shortest introns were in subgroup XIII, while the longest introns were in subgroup V. Comparable findings have been reported in Soybean [31], *Hypericum perforatum* [32] and cotton [33].

Rice and maize are monocotyledons, while common bean (*P. vulgaris*) and *Arabidopsis* are dicotyledons. Phylogenetic tree analysis revealed that Hsf members in the above four species were in the same subgroups (14) while subgroup III and XI had no monocot members, which could be attributed to evolution of plants into monocots and dicots. Hsfs of the same subgroup in these four species exhibited similar gene structures. However, the order of motifs in the four species exhibited some differences. Same subgroup Hsfs had similar motifs, such as motifs-1, 2, 3 (DBD domain) as well as motif-8 (C-terminal domain) and they also exhibited similar motif compositions, implying that Hsf members play similar functions in proteins. Comparable outcomes have been found among Hsf members in maize [34], moso bamboo (*Phyllostachys edulis*) [35], pumpkin (*Cucurbita moschata*) [36] and tea plant (*Camellia sinensis*) [37]. Moreover, Hsf members appear before plants differentiate into monocotyledons or dicotyledons, and same subgroup members exhibit similar motifs and evolutionary relationships [21].

The *cis*-elements in promoter regions of gene family members regulate the expressions of metabolic pathway-related genes [38]. In this study, seven *cis*-elements related to hormones (such as ARBE, TATC-box, P-box, AuxRR-core, TCA and TGA elements) were identified, indicating that *PvHsfs* may be involved in the roles of hormones in plant growth and development. Three stress-related *cis*-elements (LTR, ARE and MBS elements) were found in different *PvHsfs*, including *PvHsf09*, *PvHsf10*, *PvHsf15*, *PvHsf17* and *PvHsf24*. Under abiotic stress conditions, the expression levels of these *PvHsfs* exhibited significant changes (Figs. 9 and 10), confirming that stress-related *cis*-acting elements are responsive to abiotic stress. Also, Hormone-related *cis*-elements, including ABRE, AuxRR-core, P-box and TGA-elements, have been found in carnation (*Dianthus caryophyllus*) [39] and *Hypericum perforatum* [32]. Hsf members while three stress-related *cis*-elements (LTR, ARE and MBS elements) have also been found in Hsf members from *Brassica juncea* [40] and *Hypericum perforatum* [32]. Moreover, Hsf members in carnation (*Dianthus caryophyllus*) [39], *Brassica juncea* [40], *Hypericum perforatum* [32] and tea plants (*Camellia sinensis*) [37] had a CAT-box element. Under abiotic stress, the expressions of *Hsf* members exhibited some variations.

Collinearity analysis revealed that *PvHsfs* had more pairs of homologous genes in soybean (37) than in *Arabidopsis* (1) and rice (1), indicating that PvHsfs are closely associated with legumes. The collinear gene of *PvHsfs* in *Arabidopsis*, *AT2G41690*, has been reported to exert some effects during abiotic stress [41], indicating that *PvHsfs* influence abiotic stress responses in plants.

Gene expression patterns can be used to investigate the biological functions of various genes [42]. In crops, Hsf members exhibit tissue-specific expressions. For instance, *DcaHsfs* exhibit different expression patterns in carnation, as well as in members of the same class [39]. *StHsf* genes are highly expressed in various potato (*Solanum tuberosum*) tissues [43]. The expressions of *PvHsfs* in the phytozome database exhibit tissue-specificity (flowers, flower buds, young pods, leaves, green mature pods, stems and roots), indicating that *PvHsfs* are involved in plant growth and development.

During plant growth, the sprouting stage is the first and most important stage. It directly affects plant development and yield. Moreover, during abiotic stress, it is the most sensitive stage [44, 45]. As a result, studies have evaluated the effects of stress on the sprouting stage [46]. Herein, *PvHsfs* levels at the sprout stage were specific in the cotyledon and radicle, indicating that these tissues can be used as target tissues to assess *PvHsfs* at the sprout stage.

Stress, especially abiotic stress, alters the expressions of Hsf members [7]. For instance, heat stress alters the expressions of *CarHsfs* in chickpea (*Cicer arietinum*) [47]. Moreover, various stresses dysregulate the expression levels of *CsHsf* genes in tea plants [37]. In pumpkins (*Cucurbita moschata*), cold and heat stress have been shown to significantly alter the expressions of some *CmHsfs* [36]. Abiotic stressors, such as heat, cold, salt, drought and cadmium have been shown to alter the expressions of most *TaHsfs* in bread wheat (*Triticum aestivum*) [48]. Collectively, these findings imply that Hsf members can respond and resist abiotic stress. qRT-PCR assays were performed to assess the expression profiles of *PvHsfs* under heat, cold, salt and heavy metal stresses. *PvHsf01*, *PvHsf17*, *PvHsf21* and *PvHsf24* had a significant different expression under heat, cold, salt and heavy metal stress compared with CK treatment, which was similar expression-patterns in *DcaHsfs* (*D. caryophyllus*) [39]. These abiotic stressors altered the expressions of *PvHsfs*, similar to the patterns observed in other plants.

## Conclusions

In summary, this study identified 30 members of PvHsf from the reference genome and comprehensively analyzed the location, motifs, gene structure, *cis*-elements and collinearity among PvHsf members. The *PvHsfs’* expression from the phytozome database and the analysis at the sprout stage in different tissues all revealed that *PvHsfs* had the tissue-specific expression. In addition, the expression of *PvHsfs* under heat, cold, salt and heavy metal stress showed *PvHsfs* might regulate responses to abiotic stresses in common bean. This study lays the foundation for further identification of *PvHsfs* and adds to our understanding on the role of *PvHsfs* in the regulation of abiotic stress resistance in common bean.

## Materials and methods

### Identification of Hsf members in *P. vulgaris*

Genomic data (genes, cDNAs and proteins) of *P. vulgaris* (PhaVulg1_0) was derived from the ensembl plants database [49] while data of Hsf protein domain (PF00447) was obtained from the PFAM database [50]. These data were screened using the HMMER software [51] to identify Hsf members. In addition, ExPASy Proteomics Server [52] and Plant Protein Phosphorylation DataBase (P3DB) [53] were screened to identify the Hsf members in common bean (PvHsf). The location of PvHsf members was mapped based on the reference genome and named depending on their chromosomal location using TBtools [54].

### Analysis of Hsf members in *P. vulgaris*

MEGA X [55] was used to align protein sequences of PvHsf and Hsf members in three species (*Arabidopsis*, maize, rice) reported previously [11, 12, 14]. Maximum Likelihoodphy analysis was performed using 1000 replicates as bootstrap values and the jtt + g + i model predicted by MEGA. The MEME tool [56] was used to identify motifs with E-value of less than 1e^− 20^ and 10–50 amino acids numbered based on their corresponding E-values. The gene structure of *PvHsfs* was analyzed using Gene Structure Display Server (GSDS) [57]. Gene-wise [58] was used to determine the coordinates corresponding to DNA (containing exon and intron) and protein sequences. The *cis*-acting elements of *PvHsfs* were uncovered using the plantCARE software [38]. Circos software [59] was used to analyze gene duplication events in *PvHsfs* via the MCScanX function [60]. The expression of *PvHsfs* was visualized using a heatmap constructed using TBtools (phytozome data) [61]. All databases and software links are shown in Table S4.

### Preparation of plant materials and qRT-PCR analysis

A locally-grown common bean variety “Longjiang Ziyun” was obtained from the National Coarse Cereals Engineering Research Center (Daqing, Heilongjiang, China). The seeds were placed in an incubator away from light at 26 °C to allow sprouting. The plants were separately exposed to different stress treatments on the fifth day. Cold stress was induced by exposing plants to a temperature of 4 °C and heat stress was induced by exposing plants to a temperature of 45 °C [62, 63]. Salt stress was triggered by treatment with 70 mmol/L (NaCl), while heavy metal stress was simulated by exposing plants to 0.5 mg/L (CdCl_2_) and 60 mg/L (HgCl_2_) [64–66]. For control (CK) treatment, hypocotyl, radicle and cotyledon were collected as for samples in the analysis of tissue-specific expression. The radicles under abiotic stress treatments were collected as samples respectively while the CK was served as the control tissue sample. RNA Easy Fast Kit (DP452, Tiangen, Beijing) was used to extract RNA and cDNA was obtained by total RNA reverse transcription using HiScript SuperMix was used to extract for qPCR (+gDNA wiper) (R223–01, Vazyme, Nanjing). The Primer premier 5.0 software (PREMIER Biosoft, San Francisco, USA) was used to design primers of PvHsf members (Table S5). In this experiment, *Pvactin11* gene served as the internal reference gene [67]. The expression of each PvHsf member was determined through qRT-PCR using the Light Cycler system (Roche 480II, Roche, Switzerland) and *TransStart*® Top Green qPCR SuperMix (AQ131–04, TransGen Biotech, Beijing). For each treatment, three biological replicates were prepared, and for each sample, three technical replicates were prepared. The relative mRNA expression was calculated as previously described [68].

## Supplementary Information

**Additional file 1: Figure S1.** The Motif structure (Motif1-Motif10) of PvHsf members.

**Additional file 2: Figure S2.** The Motif structure (Motif1-Motif20) of Hsf members in *Arabidopsis*, rice (*Oryza sativa*), maize (*Zea mays*) and common bean (*Phaseolus vulgaris*).

**Additional file 3: Table S1.** Identification of PvHsf members in *P. vulgaris.*

**Additional file 4: Table S2.** The *cis*-acting elements of *PvHsfs*.

**Additional file 5: Table S3.** KA/KS of *PvHsfs*.

**Additional file 6: Table S4.** The database and software websites.

**Additional file 7: Table S5.** QRT-PCR primer of *PvHsfs* designed by Primer premier 5.0 software.

## Data Availability

All data generated or analysed during this study are included in this published article and its supplementary information files.

## Abbreviations

Hsf: Heat shock transcription factor.
qRT-PCR: Quantitative Real-time PCR
Ka/Ks: The ratio of the number of nonsynonymous substitutions per nonsynonymous site (Ka) to the number of synonymous substitutions per synonymous site (Ks)

## References

[1] Nichols NN, Sutivisedsak N, Dien BS, Biswas A, Lesch WC, Cotta MA. Conversion of starch from dry common beans (*Phaseolus vulgaris* L.) to ethanol. Ind Crop Prod. 2011. 10.1016/j.indcrop.2010.12.029.

[2] Lee SC, Gepts PL, Whitaker JR. Protein structures of common bean (*Phaseolus vulgaris*) alpha-amylase inhibitors. J Agric Food Chem. 2002. 10.1021/jf020189t. 10.1021/jf020189t 12381161

[3] Singh RS, Walia AK. Microbial lectins and their prospective mitogenic potential. Crit Rev Microbiol. 2014. 10.3109/1040841x.2012.733680. 10.3109/1040841X.2012.733680 23215777

[4] Delgado-Salinas A, Turley T, Richman A, Lavin M. Phylogenetic analysis of the cultivated and wild species of Phaseolus (Fabaceae). Syst Bot. 1999. 10.2307/2419699.

[5] Ohama N, Sato H, Shinozaki K, Yamaguchi-Shinozaki K. Transcriptional regulatory network of plant heat stress response. Trends Plant Sci. 2017. 10.1016/j.tplants.2016.08.015. 10.1016/j.tplants.2016.08.015 27666516

[6] Scharf KD, Rose S, Zott W, Schöffl F, Nover L. Three tomato genes code for heat stress transcription factors with a region of remarkable homology to the DNA-binding domain of the yeast HSF. EMBO J. 1990. 10.1002/j.1460-2075.1990.tb07900.x. 10.1002/j.1460-2075.1990.tb07900.x PMC552242 2148291

[7] Scharf KD, Berberich T, Ebersberger I, Nover L. The plant heat stress transcription factor (Hsf) family: structure, function and evolution. Biochim Biophys Acta. 2012. 10.1016/j.bbagrm.2011.10.002. 10.1016/j.bbagrm.2011.10.002 22033015

[8] Wu C. Heat shock transcription factors: structure and regulation. Annu Rev Cell Dev Biol. 1995. 10.1146/annurev.cb.11.110195.002301. 10.1146/annurev.cb.11.110195.002301 8689565

[9] Fragkostefanakis S, Röth S, Schleiff E, Scharf KD. Prospects of engineering thermotolerance in crops through modulation of heat stress transcription factor and heat shock protein networks. Plant Cell Environ. 2015. 10.1111/pce.12396. 10.1111/pce.12396 24995670

[10] Bharti K, Von Koskull-Döring P, Bharti S, Kumar P, Tintschl-Körbitzer A, Treuter E, et al. Tomato heat stress transcription factor HsfB1 represents a novel type of general transcription coactivator with a histone-like motif interacting with the plant CREB binding protein ortholog HAC1. Plant Cell. 2004. 10.1105/tpc.019927. 10.1105/tpc.019927 PMC490043 15131252

[11] Busch W, Wunderlich M, Schöffl F. Identification of novel heat shock factor-dependent genes and biochemical pathways in *Arabidopsis thaliana*. Plant J. 2005. 10.1111/j.1365-313X.2004.02272.x. 10.1111/j.1365-313X.2004.02272.x 15610345

[12] Guo J, Wu J, Ji Q, Wang C, Luo L, Yuan Y, et al. Genome-wide analysis of heat shock transcription factor families in rice and *Arabidopsis*. J Genet Genomics. 2008. 10.1016/s1673-8527(08)60016-8. 10.1016/S1673-8527(08)60016-8 18407058

[13] Chung E, Kim KM, Lee JH. Genome-wide analysis and molecular characterization of heat shock transcription factor family in *Glycine max*. J Genet Genomics. 2013. 10.1016/j.jgg.2012.12.002. 10.1016/j.jgg.2012.12.002 23522385

[14] Lin YX, Jiang HY, Chu ZX, Tang XL, Zhu SW, Cheng BJ. Genome-wide identification, classification and analysis of heat shock transcription factor family in maize. BMC Genomics. 2011. 10.1186/1471-2164-12-76. 10.1186/1471-2164-12-76 PMC3039612 21272351

[15] Prieto-Dapena P, Almoguera C, Personat JM, Merchan F, Jordano J. Seed-specific transcription factor HSFA9 links late embryogenesis and early photomorphogenesis. J Exp Bot. 2017. 10.1093/jxb/erx020. 10.1093/jxb/erx020 PMC5441851 28207924

[16] Lin Q, Jiang Q, Lin J, Wang D, Li S, Liu C, et al. Heat shock transcription factors expression during fruit development and under hot air stress in Ponkan (*Citrus reticulata* Blanco cv. Ponkan) fruit. Gene. 2015. 10.1016/j.gene.2015.01.024. 10.1016/j.gene.2015.01.024 25596345

[17] Liu M, Ma Z, Zheng T, Wang J, Huang L, Sun W, et al. The potential role of Auxin and Abscisic acid balance and *FtARF*2 in the final size determination of Tartary buckwheat fruit. Int J Mol Sci. 2018. 10.3390/ijms19092755. 10.3390/ijms19092755 PMC6163771 30217096

[18] Huang Y, Li MY, Wang F, Xu ZS, Huang W, Wang GL, et al. Heat shock factors in carrot: genome-wide identification, classification, and expression profiles response to abiotic stress. Mol Biol Rep. 2015. 10.1007/s11033-014-3826-x. 10.1007/s11033-014-3826-x 25403331

[19] Huang YC, Niu CY, Yang CR, Jinn TL. The heat stress factor HSFA6b connects ABA signaling and ABA-mediated heat responses. Plant Physiol. 2016. 10.1104/pp.16.00860. 10.1104/pp.16.00860 PMC5047099 27493213

[20] Zhang J, Jia H, Li J, Li Y, Lu M, Hu J. Molecular evolution and expression divergence of the *Populus euphratica Hsf* genes provide insight into the stress acclimation of desert poplar. Sci Rep. 2016. 10.1038/srep30050. 10.1038/srep30050 PMC4948027 27425424

[21] Liu M, Huang Q, Sun W, Ma Z, Huang L, Wu Q, et al. Genome-wide investigation of the heat shock transcription factor (*Hsf*) gene family in Tartary buckwheat (*Fagopyrum tataricum*). BMC Genomics. 2019. 10.1186/s12864-019-6205-0. 10.1186/s12864-019-6205-0 PMC6858736 31730445

[22] Wang F, Dong Q, Jiang H, Zhu S, Chen B, Xiang Y. Genome-wide analysis of the heat shock transcription factors in *Populus trichocarpa* and *Medicago truncatula*. Mol Biol Rep. 2012. 10.1007/s11033-011-0933-9. 10.1007/s11033-011-0933-9 21625849

[23] Nover L, Bharti K, Döring P, Mishra SK, Ganguli A, Scharf KD. *Arabidopsis* and the heat stress transcription factor world: how many heat stress transcription factors do we need? Cell Stress Chaperones. 2001. https://doi.org/10.1379/1466-1268(2001)006<0177:aathst>2.0.co;2. 10.1379/1466-1268(2001)006<0177:aathst>2.0.co;2 PMC434399 11599559

[24] Liu G, Chai F, Wang Y, Jiang J, Duan W, Wang Y, et al. Genome-wide identification and classification of HSF family in grape, and their transcriptional analysis under heat acclimation and heat stress. Horticult Plant J. 2018. 10.1016/j.hpj.2018.06.001.

[25] Davletova S, Rizhsky L, Liang H, Shengqiang Z, Oliver DJ, Coutu J, et al. Cytosolic ascorbate peroxidase 1 is a central component of the reactive oxygen gene network of Arabidopsis. Plant Cell. 2005. 10.1105/tpc.104.026971. 10.1105/tpc.104.026971 PMC544504 15608336

[26] Zhang J, Liu B, Li J, Zhang L, Wang Y, Zheng H, et al. *Hsf* and *Hsp* gene families in *Populus*: genome-wide identification, organization and correlated expression during development and in stress responses. BMC Genomics. 2015. 10.1186/s12864-015-1398-3. 10.1186/s12864-015-1398-3 PMC4373061 25887520

[27] Wang J, Sun N, Deng T, Zhang L, Zuo K. Genome-wide cloning, identification, classification and functional analysis of cotton heat shock transcription factors in cotton (*Gossypium hirsutum*). BMC Genomics. 2014. 10.1186/1471-2164-15-961. 10.1186/1471-2164-15-961 PMC4233062 25378022

[28] Tan B, Yan L, Li H, Lian X, Cheng J, Wang W, et al. Genome-wide identification of HSF family in peach and functional analysis of *PpHSF5* involvement in root and aerial organ development. PeerJ. 2021. 10.7717/peerj.10961. 10.7717/peerj.10961 PMC7958895 33763299

[29] Shaul O. How introns enhance gene expression. Int J Biochem Cell Biol. 2017. 10.1016/j.biocel.2017.06.016. 10.1016/j.biocel.2017.06.016 28673892

[30] Li Y, Chen D, Luo S, Zhu Y, Jia X, Duan Y, et al. Intron-mediated regulation of β-tubulin genes expression affects the sensitivity to carbendazim in *Fusarium graminearum*. Curr Genet. 2019. 10.1007/s00294-019-00960-4. 10.1007/s00294-019-00960-4 30941494

[31] Li PS, Yu TF, He GH, Chen M, Zhou YB, Chai SC, et al. Genome-wide analysis of the Hsf family in soybean and functional identification of *GmHsf-34* involvement in drought and heat stresses. BMC Genomics. 2014. 10.1186/1471-2164-15-1009. 10.1186/1471-2164-15-1009 PMC4253008 25416131

[32] Zhou L, Yu X, Wang D, Li L, Zhou W, Zhang Q, et al. Genome-wide identification, classification and expression profile analysis of the HSF gene family in *Hypericum perforatum*. PeerJ. 2021. 10.7717/peerj.11345. 10.7717/peerj.11345 PMC8106910 33996286

[33] Rehman A, Atif RM, Azhar MT, Peng Z, Li H, Qin G, et al. X., genome wide identification, classification and functional characterization of heat shock transcription factors in cultivated and ancestral cottons (*Gossypium* spp.). Int J Biol Macromol. 2021. 10.1016/j.ijbiomac.2021.05.016. 10.1016/j.ijbiomac.2021.05.016 33965497

[34] Zhang H, Li G, Fu C, Duan S, Hu D, Guo X. Genome-wide identification, transcriptome analysis and alternative splicing events of Hsf family genes in maize. Sci Rep. 2020. 10.1038/s41598-020-65068-z. 10.1038/s41598-020-65068-z PMC7229205 32415117

[35] Huang B, Huang Z, Ma R, Chen J, Zhang Z, Yrjälä K. Genome-wide identification and analysis of the heat shock transcription factor family in moso bamboo (*Phyllostachys edulis*). Sci Rep. 2021. 10.1038/s41598-021-95899-3. 10.1038/s41598-021-95899-3 PMC8363633 34389742

[36] Shen C, Yuan J. Genome-wide characterization and expression analysis of the heat shock transcription factor family in pumpkin (*Cucurbita moschata*). BMC Plant Biol. 2020. 10.1186/s12870-020-02683-y. 10.1186/s12870-020-02683-y PMC7557022 33054710

[37] Zhang X, Xu W, Ni D, Wang M, Guo G. Genome-wide characterization of tea plant (*Camellia sinensis*) Hsf transcription factor family and role of *CsHsfA2* in heat tolerance. BMC Plant Biol. 2020. 10.1186/s12870-020-02462-9. 10.1186/s12870-020-02462-9 PMC7260767 32471355

[38] Lescot M, Déhais P, Thijs G, Marchal K, Moreau Y, Van de Peer Y, et al. PlantCARE, a database of plant *cis*-acting regulatory elements and a portal to tools for in silico analysis of promoter sequences. Nucleic Acids Res. 2002. 10.1093/nar/30.1.325. 10.1093/nar/30.1.325 PMC99092 11752327

[39] Li W, Wan XL, Yu JY, Wang KL, Zhang J. Genome-wide identification, classification, and expression analysis of the *Hsf* gene family in carnation (*Dianthus caryophyllus*). Int J Mol Sci. 2019. 10.3390/ijms20205233. 10.3390/ijms20205233 PMC6829504 31652538

[40] Li M, Xie F, Li Y, Gong L, Luo Y, Zhang Y, et al. Genome-wide analysis of the heat shock transcription factor gene family in *Brassica juncea*: structure, evolution, and expression profiles. DNA Cell Biol. 2020. 10.1089/dna.2020.5922. 10.1089/dna.2020.5922 32945687

[41] Sarry JE, Kuhn L, Ducruix C, Lafaye A, Junot C, Hugouvieux V, et al. The early responses of *Arabidopsis thaliana* cells to cadmium exposure explored by protein and metabolite profiling analyses. Proteomics. 2006. 10.1002/pmic.200500543. 10.1002/pmic.200500543 16502469

[42] Maheswari U, Jabbari K, Petit JL, Porcel BM, Allen AE, Cadoret JP, et al. Digital expression profiling of novel diatom transcripts provides insight into their biological functions. Genome Biol. 2010. 10.1186/gb-2010-11-8-r85. 10.1186/gb-2010-11-8-r85 PMC2945787 20738856

[43] Tang R, Zhu W, Song X, Lin X, Cai J, Wang M, et al. Genome-wide identification and function analyses of heat shock transcription factors in potato. Front Plant Sci. 2016. 10.3389/fpls.2016.00490. 10.3389/fpls.2016.00490 PMC4836240 27148315

[44] Fahad S, Bajwa AA, Nazir U, Anjum SA, Farooq A, Zohaib A, et al. Crop production under drought and heat stress: plant responses and management options. Front Plant Sci. 2017. 10.3389/fpls.2017.01147. 10.3389/fpls.2017.01147 PMC5489704 28706531

[45] Ibrahim EA. Seed priming to alleviate salinity stress in germinating seeds. J Plant Physiol. 2016. 10.1016/j.jplph.2015.12.011. 10.1016/j.jplph.2015.12.011 26812088

[46] Yadav PV, Kumari M, Ahmed Z. Seed priming mediated germination improvement and tolerance to subsequent exposure to cold and salt stress in Capsicum. Res J Seed Sci. 2011. 10.3923/rjss.2011.125.136.

[47] Chidambaranathan P, Jagannadham PTK, Satheesh V, Kohli D, Basavarajappa SH, Chellapilla B, et al. Genome-wide analysis identifies chickpea (*Cicer arietinum*) heat stress transcription factors (*Hsfs*) responsive to heat stress at the pod development stage. J Plant Res. 2018. 10.1007/s10265-017-0948-y. 10.1007/s10265-017-0948-y 28474118

[48] Zhou M, Zheng S, Liu R, Lu J, Lu L, Zhang C, et al. Genome-wide identification, phylogenetic and expression analysis of the heat shock transcription factor family in bread wheat (*Triticum aestivum* L.). BMC Genomics. 2019. 10.1186/s12864-019-5876-x. 10.1186/s12864-019-5876-x PMC6580518 31215411

[49] Zhang Q, Zhang WJ, Yin ZG, Li WJ, Zhao HH, Zhang S, et al. Genome- and Transcriptome-wide identification of C3Hs in common bean (*Phaseolus vulgaris* L.) and structural and expression-based analyses of their functions during the sprout stage under salt-stress conditions. Front Genet. 2020. 10.3389/fgene.2020.564607. 10.3389/fgene.2020.564607 PMC7522512 33101386

[50] Finn RD, Bateman A, Clements J, Coggill P, Eberhardt RY, Eddy SR, et al. Pfam: the protein families database. Nucleic Acids Res. 2014. 10.1093/nar/gkt1223. 10.1093/nar/gkt1223 PMC3965110 24288371

[51] Finn RD, Clements J, Arndt W, Miller BL, Wheeler TJ, Schreiber F, et al. HMMER web server: 2015 update. Nucleic Acids Res. 2015. 10.1093/nar/gkv397. 10.1093/nar/gkv397 PMC4489315 25943547

[52] Hoogland C, Mostaguir K, Appel RD, Lisacek F. The world-2DPAGE constellation to promote and publish gel-based proteomics data through the ExPASy server. J Proteome. 2008. 10.1016/j.jprot.2008.02.005. 10.1016/j.jprot.2008.02.005 18617148

[53] Yao Q, Xu D. Bioinformatics analysis of protein phosphorylation in plant systems biology using P3DB. Methods Mol Biol. 2017. 10.1007/978-1-4939-6783-4_6. 10.1007/978-1-4939-6783-4_6 28150236

[54] Chen C, Chen H, Zhang Y, Thomas HR, Frank MH, He Y, et al. TBtools: an integrative toolkit developed for interactive analyses of big biological data. Mol Plant. 2020. 10.1016/j.molp.2020.06.009. 10.1016/j.molp.2020.06.009 32585190

[55] Kumar S, Stecher G, Li M, Knyaz C, Tamura K. MEGA X: molecular evolutionary genetics analysis across computing platforms. Mol Biol Evol. 2018. 10.1093/molbev/msy096. 10.1093/molbev/msy096 PMC5967553 29722887

[56] Bailey TL, Boden M, Buske FA, Frith M, Grant CE, Clementi L, et al. MEME SUITE: tools for motif discovery and searching. Nucleic Acids Res. 2009. 10.1093/nar/gkp335. 10.1093/nar/gkp335 PMC2703892 19458158

[57] Hu B, Jin J, Guo AY, Zhang H, Luo J, Gao G. GSDS 2.0: an upgraded gene feature visualization server. Bioinformatics. 2015. 10.1093/bioinformatics/btu817. 10.1093/bioinformatics/btu817 PMC4393523 25504850

[58] Simmons MP, Sloan DB, Springer MS, Gatesy J. Gene-wise resampling outperforms site-wise resampling in phylogenetic coalescence analyses. Mol Phylogenet Evol. 2019. 10.1016/j.ympev.2018.10.001. 10.1016/j.ympev.2018.10.001 30391518

[59] Krzywinski M, Schein J, Birol I, Connors J, Gascoyne R, Horsman D, et al. Circos: an information aesthetic for comparative genomics. Genome Res. 2009. 10.1101/gr.092759.109. 10.1101/gr.092759.109 PMC2752132 19541911

[60] Wang Y, Li J, Paterson AH. *MCScanX-transposed*: detecting transposed gene duplications based on multiple colinearity scans. Bioinformatics. 2013. 10.1093/bioinformatics/btt150. 10.1093/bioinformatics/btt150 23539305

[61] Goodstein DM, Shu S, Howson R, Neupane R, Hayes RD, Fazo J, et al. Phytozome: a comparative platform for green plant genomics. Nucleic Acids Res. 2012. 10.1093/nar/gkr944. 10.1093/nar/gkr944 PMC3245001 22110026

[62] Alsamir M, Mahmood T, Trethowan R, Ahmad N. An overview of heat stress in tomato (Solanum lycopersicum L.). Saudi. J Biol Sci. 2021. 10.1016/j.sjbs.2020.11.088. 10.1016/j.sjbs.2020.11.088 PMC7938145 33732051

[63] Wang F, Chen X, Dong S, Jiang X, Wang L, Yu J, et al. Crosstalk of PIF4 and DELLA modulates CBF transcript and hormone homeostasis in cold response in tomato. Plant Biotechnol J. 2020. 10.1111/pbi.13272. 10.1111/pbi.13272 PMC7061876 31584235

[64] Mohammadi S, Pourakbar L, Siavash Moghaddam S, Popović-Djordjević J. The effect of EDTA and citric acid on biochemical processes and changes in phenolic compounds profile of okra (*Abelmoschus esculentus* L.) under mercury stress. Ecotoxicol Environ Saf. 2021. 10.1016/j.ecoenv.2020.111607. 10.1016/j.ecoenv.2020.111607 33396127

[65] Zhang Q, Zhang W-j, Yin Z-g, Li W-j, Xia C-Y, Sun H-Y, et al. Genome-wide identification reveals the potential functions of the bZIP gene family in common bean (*Phaseolus vulgaris*) in response to salt stress during the sprouting stage. J Plant Growth Regul. 2021. 10.1007/s00344-021-10497-x.

[66] Zhao Q, Wang H, Du Y, Rogers HJ, Wu Z, Jia S, et al. MSH2 and MSH6 in mismatch repair system account for soybean (*Glycine max* (L.) Merr.) tolerance to cadmium toxicity by determining DNA damage response. J Agric Food Chem. 2020. 10.1021/acs.jafc.9b06599. 10.1021/acs.jafc.9b06599 31971785

[67] Zhang Q, Li M, Xia CY, Zhang WJ, Yin ZG, Zhang YL, et al. Transcriptome-based analysis of salt-related genes during the sprout stage of common bean (*Phaseolus vulgaris*) under salt stress conditions. Biotechnol Biotechnol Equip. 2021. 10.1080/13102818.2021.1954091.

[68] Livak KJ, Schmittgen TD. Analysis of relative gene expression data using real-time quantitative PCR and the 2(−ΔΔC(T)) method. Methods. 2001. 10.1006/meth.2001.1262. 10.1006/meth.2001.1262 11846609

