# Supplementary figures and images for: Heat shock transcription factor (Hsf) gene family in common bean (Phaseolus vulgaris): genome-wide identification, phylogeny, evolutionary expansion and expression analyses at the sprout stage under abiotic stress
Source: BMC Plant Biol. 2022 Jan 14;22:33. doi: 10.1186/s12870-021-03417-4 (PMC8759166; doi:10.1186/s12870-021-03417-4)

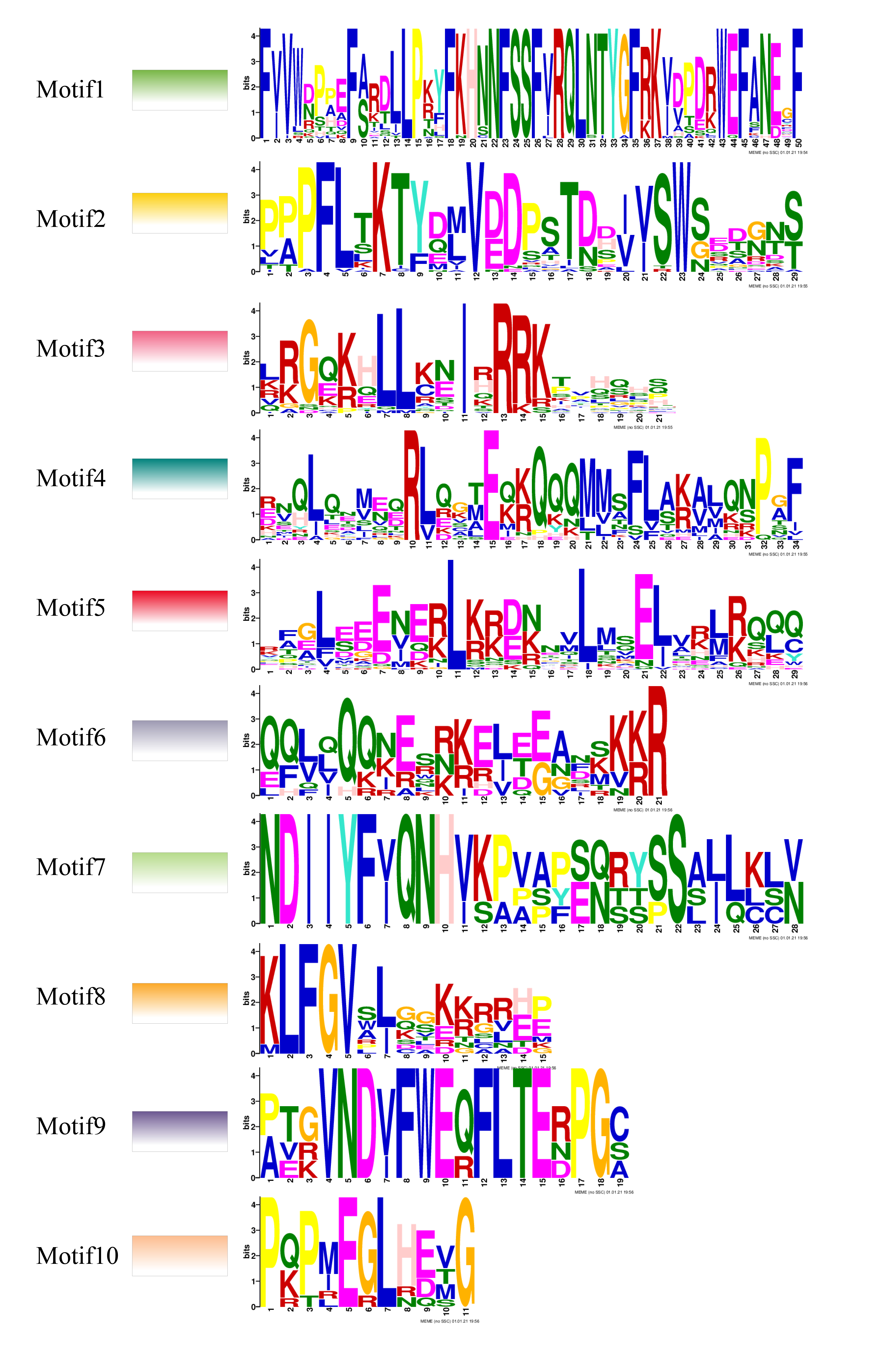

Supplement: Supplementary file 1 — Additional file 1: Figure S1. The Motif structure (Motif1-Motif10) of PvHsf members. [file 12870_2021_3417_MOESM1_ESM.jpg]

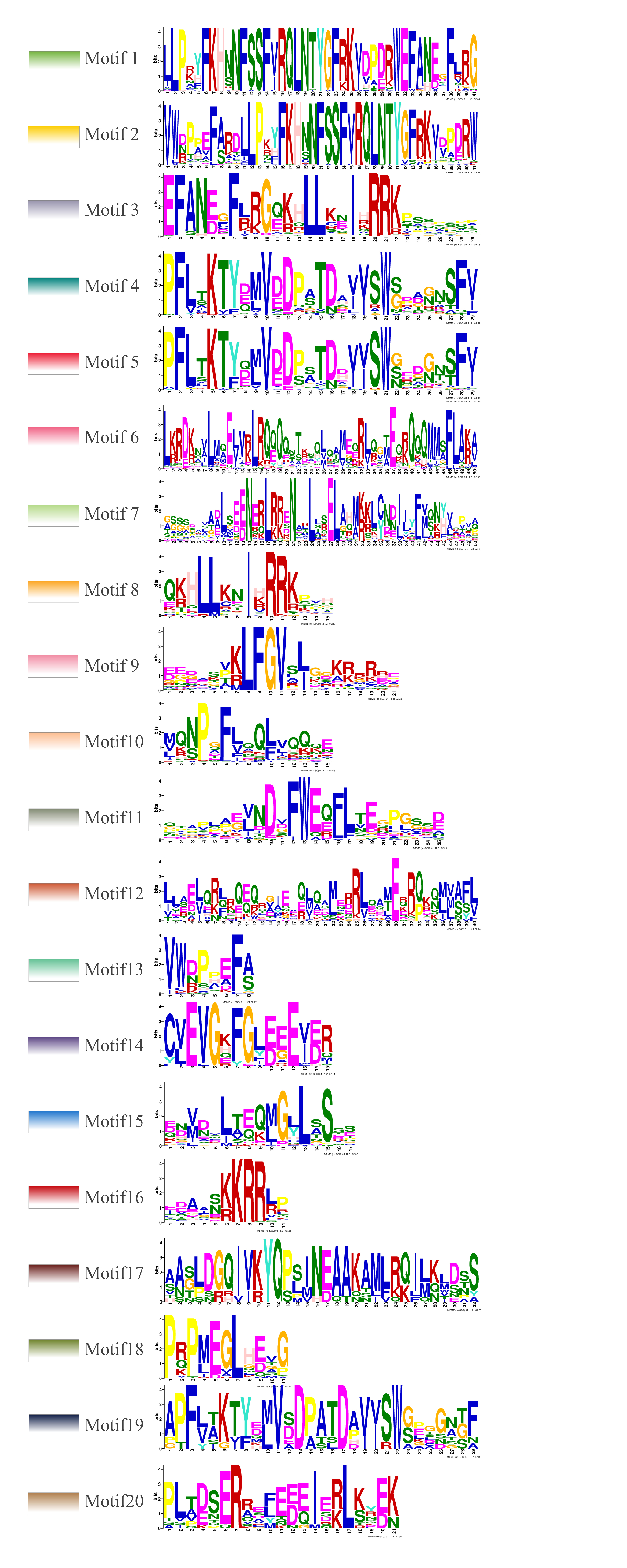

Supplement: Supplementary file 2 — Additional file 2: Figure S2. The Motif structure (Motif1-Motif20) of Hsf members in Arabidopsis, rice (Oryza sativa), maize (Zea mays) and common bean (Phaseolus vulgaris). [file 12870_2021_3417_MOESM2_ESM.jpg]
