# Supplementary material for: Heat shock transcription factor (Hsf) gene family in common bean (Phaseolus vulgaris): genome-wide identification, phylogeny, evolutionary expansion and expression analyses at the sprout stage under abiotic stress
Source: BMC Plant Biol. 2022 Jan 14;22:33. doi: 10.1186/s12870-021-03417-4 (PMC8759166; doi:10.1186/s12870-021-03417-4)
Supplement: Supplementary file 3 — Additional file 3: Table S1. Identification of PvHsf members in P. vulgaris. [file 12870_2021_3417_MOESM3_ESM.docx]

**Table S1:** Identification of PvHsf members in *P. vulgaris.*

| Gene name | Gene Bank | Gene_id | Chr | Location | Protein length | CDS length | Isoelectric point | Molecular weigth | Instability index | Aliphatic index |
| --- | --- | --- | --- | --- | --- | --- | --- | --- | --- | --- |
| *PvHsf01* | ESW32855 | *Phvul.001G022900* | 1 | 1996915:1998671 | 339 | 1017 | 7.31 | 38843.55 | 56.91 | 72.09 |
| *PvHsf02* | ESW33023 | *Phvul.001G037000* | 1 | 3563513:3566108 | 373 | 1119 | 4.91 | 42462.58 | 57.05 | 70.8 |
| *PvHsf03* | ESW34174 | *Phvul.001G131000* | 1 | 37103243:37105710 | 232 | 696 | 9.08 | 26585.97 | 58.18 | 60.52 |
| *PvHsf04* | ESW34460 | *Phvul.001G154700* | 1 | 41183051:41185997 | 366 | 1098 | 5.54 | 42242.3 | 62.17 | 66.01 |
| *PvHsf05* | ESW35805 | *Phvul.001G266300* | 1 | 51913229:51915272 | 441 | 1323 | 5.61 | 49829.8 | 58.12 | 73.2 |
| *PvHsf06* | ESW28800 | *Phvul.002G019100* | 2 | 2097022:2099089 | 284 | 852 | 8.44 | 31522.5 | 42.72 | 65.18 |
| *PvHsf07* | ESW29066 | *Phvul.002G040500* | 2 | 3867113:3870724 | 465 | 1395 | 5.26 | 51726.58 | 53.25 | 72.65 |
| *PvHsf08* | ESW30265 | *Phvul.002G138500* | 2 | 27124383:27128271 | 490 | 1470 | 5.02 | 54230.43 | 60.77 | 68.43 |
| *PvHsf09* | ESW30466 | *Phvul.002G155300* | 2 | 29686274:29687420 | 357 | 1071 | 4.93 | 39048.5 | 53.65 | 74.62 |
| *PvHsf10* | ESW31317 | *Phvul.002G228400* | 2 | 39319738:39321519 | 206 | 618 | 7.61 | 23907.29 | 54 | 68.16 |
| *PvHsf11* | ESW31485 | *Phvul.002G242000* | 2 | 40885716:40887971 | 403 | 1209 | 5.14 | 45804.06 | 52.46 | 70.67 |
| *PvHsf12* | ESW32438 | *Phvul.002G322700* | 2 | 48161637:48164747 | 360 | 1080 | 4.9 | 41209.45 | 53.07 | 79.31 |
| *PvHsf13* | ESW26630 | *Phvul.003G135200* | 3 | 32657019:32659341 | 404 | 1212 | 5.83 | 46423.45 | 47.77 | 64.58 |
| *PvHsf14* | ESW26632 | *Phvul.003G135400* | 3 | 32680148:32682497 | 404 | 1212 | 5.96 | 46308.2 | 45.99 | 63.86 |
| *PvHsf15* | ESW27920 | *Phvul.003G244000* | 3 | 46868205:46871391 | 278 | 834 | 5.92 | 30720.38 | 32.54 | 65.94 |
| *PvHsf16* | ESW28397 | *Phvul.003G283300* | 3 | 50951530:50955595 | 474 | 1422 | 4.9 | 52777.74 | 58.2 | 67.66 |
| *PvHsf17* | ESW24312 | *Phvul.004G119600* | 4 | 38764226:38766008 | 340 | 1020 | 5.62 | 37155.59 | 52.21 | 68.88 |
| *PvHsf18* | ESW24823 | *Phvul.004G163300* | 4 | 44509237:44511021 | 317 | 951 | 5.95 | 35402.07 | 55.6 | 74.16 |
| *PvHsf19* | ESW18134 | *Phvul.006G015700* | 6 | 7459944:7461399 | 269 | 807 | 6.56 | 31488.57 | 53.23 | 75.69 |
| *PvHsf20* | ESW18356 | *Phvul.006G034200* | 6 | 13922202:13927066 | 487 | 1461 | 5.44 | 54538.32 | 65.12 | 66.78 |
| *PvHsf21* | ESW19988 | *Phvul.006G171700* | 6 | 28240620:28242883 | 386 | 1158 | 5.01 | 43876.73 | 55.98 | 73.42 |
| *PvHsf22* | ESW15308 | *Phvul.007G061800* | 7 | 5381401:5383424 | 353 | 1059 | 5.29 | 40995.34 | 46.89 | 81.9 |
| *PvHsf23* | ESW15375 | *Phvul.007G067800* | 7 | 6095460:6096981 | 304 | 912 | 7.02 | 34047.3 | 59.53 | 70.92 |
| *PvHsf24* | ESW16658 | *Phvul.007G174800* | 7 | 40921790:40924438 | 357 | 1071 | 5.33 | 41497.85 | 55.46 | 73.98 |
| *PvHsf25* | ESW17249 | *Phvul.007G223400* | 7 | 46292132:46295237 | 452 | 1356 | 4.83 | 51271.3 | 55.85 | 71.99 |
| *PvHsf26* | ESW17589 | *Phvul.007G251900* | 7 | 49052994:49056187 | 223 | 669 | 8.14 | 25943.55 | 47.98 | 74.26 |
| *PvHsf27* | ESW17906 | *Phvul.007G278200* | 7 | 51513590:51515480 | 334 | 1002 | 4.82 | 38667.39 | 65.17 | 81.65 |
| *PvHsf28* | ESW13508 | *Phvul.008G202500* | 8 | 51238104:51239527 | 269 | 807 | 7.22 | 31202.45 | 63.08 | 73.94 |
| *PvHsf29* | ESW08686 | *Phvul.009G065800* | 9 | 11250307:11251969 | 364 | 1092 | 8.44 | 40835.8 | 48.52 | 66.68 |
| *PvHsf30* | ESW08828 | *Phvul.009G078300* | 9 | 12751693:12753965 | 373 | 1119 | 4.9 | 41688.35 | 58.85 | 80.99 |
