# Supplementary material for: Heat shock transcription factor (Hsf) gene family in common bean (Phaseolus vulgaris): genome-wide identification, phylogeny, evolutionary expansion and expression analyses at the sprout stage under abiotic stress
Source: BMC Plant Biol. 2022 Jan 14;22:33. doi: 10.1186/s12870-021-03417-4 (PMC8759166; doi:10.1186/s12870-021-03417-4)
Supplement: Supplementary file 4 — Additional file 4: Table S2. The cis-acting elements of PvHsfs. [file 12870_2021_3417_MOESM4_ESM.docx]

**Table S2:** The *cis*-acting elements of *PvHsfs.*

| Element | Core sequence | Function |
| --- | --- | --- |
| TCA-element | CCATCTTTTT | *cis*-acting element involved in salicylic acid responsiveness. |
| CAT-box | GCCACT | cis-acting regulatory element related to meristem expression. |
| TGA-element | AACGAC | auxin-responsive element. |
| ARE | AAACCA | cis-acting regulatory element essential for the anaerobic induction. |
| LTR | CCGAAA | cis-acting element involved in low-temperature responsiveness. |
| P-box | CAACAAACCCCTT | gibberellin-responsive element and part of a light responsive element. |
| ABRE | ACGTG | cis-acting element involved in the abscisic acid responsiveness. |
| MBS | CAACTG | MYB binding site involved in drought-inducibility. |
| TATC-box | TATCCCA | cis-acting element involved in gibberellin-responsiveness. |
| AuxRR-core | GGTCCAT | cis-acting regulatory element involved in auxin responsiveness. |
