# Supplementary material for: Heat shock transcription factor (Hsf) gene family in common bean (Phaseolus vulgaris): genome-wide identification, phylogeny, evolutionary expansion and expression analyses at the sprout stage under abiotic stress
Source: BMC Plant Biol. 2022 Jan 14;22:33. doi: 10.1186/s12870-021-03417-4 (PMC8759166; doi:10.1186/s12870-021-03417-4)
Supplement: Supplementary file 5 — Additional file 5: Table S3. KA/KS of PvHsfs. [file 12870_2021_3417_MOESM5_ESM.docx]

**Table S3:** KA/KS of *PvHsfs.*

| Seq_1 | Seq_2 | Ka | Ks | Ka/Ks |
| --- | --- | --- | --- | --- |
| PvHsf22 | PvHsf19 | 0.146526766 | 0.794641852 | 0.184393467 |
| PvHsf13 | PvHsf14 | 0.023497828 | 0.061258523 | 0.383584629 |
