# Supplementary material for: Heat shock transcription factor (Hsf) gene family in common bean (Phaseolus vulgaris): genome-wide identification, phylogeny, evolutionary expansion and expression analyses at the sprout stage under abiotic stress
Source: BMC Plant Biol. 2022 Jan 14;22:33. doi: 10.1186/s12870-021-03417-4 (PMC8759166; doi:10.1186/s12870-021-03417-4)
Supplement: Supplementary file 6 — Additional file 6: Table S4. The database and software websites. [file 12870_2021_3417_MOESM6_ESM.docx]

**Table S4.** The database and software websites

| Database or software | URL |
| --- | --- |
| Pfam | http://pfam.sanger.ac.uk/ |
| HMMER | http://hmmer.janelia.org/ |
| InterPro | http://www.ebi.ac.uk/interpro/ |
| SMART | http://smart.embl-heidelberg.de/ |
| WoLF PSORT | https://wolfpsort.hgc.jp/ |
| ExPASy Proteomics Server | http://prosite.expasy.org/ |
| P3DB | http://www.p3db.org/ |
| GSDS platform | http://gsds.cbi.pku.edu.cn/ |
| MEME tool | http://meme.nbcr.net/meme/ |
| circos | http://circos.ca/ |
| phytozome | https://phytozome.jgi.doe.gov/pz/portal.html |
