# Supplementary material for: Heat shock transcription factor (Hsf) gene family in common bean (Phaseolus vulgaris): genome-wide identification, phylogeny, evolutionary expansion and expression analyses at the sprout stage under abiotic stress
Source: BMC Plant Biol. 2022 Jan 14;22:33. doi: 10.1186/s12870-021-03417-4 (PMC8759166; doi:10.1186/s12870-021-03417-4)
Supplement: Supplementary file 7 — Additional file 7: Table S5. QRT-PCR primer of PvHsfs designed by Primer premier 5.0 software. [file 12870_2021_3417_MOESM7_ESM.docx]

**Table S5:** QRT-PCR primer of *PvHsfs* designed by Primer premier 5.0 software.

| Primer | Sequence (5 'to 3') | Primer | Sequence (5 'to 3') |
| --- | --- | --- | --- |
| *PvHsf01F* | GATTGTGCCTGATAGGTGGG | *PvHsf01R* | TGATGCTGCTGATGGGTTTT |
| *PvHsf03F* | TTCTGGCTACACTACTCTGG | *PvHsf03R* | AATCAAGCAGTTCCTTACAC |
| *PvHsf05F* | CGTTCCTACTGAAGACATAC | *PvHsf05R* | GCAAACTCCCATCGC |
| *PvHsf09F* | CGAGTTTCATCGTTTGGC | *PvHsf09R* | TTCCGAAATCCATAGGTGT |
| *PvHsf10F* | AAACGAAGGTTACCGAGA | *PvHsf10R* | CCACGGACGATTCAAG |
| *PvHsf15F* | CCATTTTTGACGAAGACG | *PvHsf15R* | TTTGAAGTATTTGGGAAG |
| *PvHsf16F* | TGTTCAGAGCCCTGGTTT | *PvHsf16R* | ATTTGCCTAACCATTGCT |
| *PvHsf17F* | CGGTTGTTGTTCCGACGC | *PvHsf17R* | GCGAGCGACGGTGAAGAG |
| *PvHsf21F* | TTCCTGTGGCTACTGGTGTT | *PvHsf21R* | CCTATCTGTTCTGGAGGGTTATT |
| *PvHsf22F* | AAGGGTTTCTCAGAGGACAA | *PvHsf22R* | TGGTCTTTTGTGTTCTGCTG |
| *PvHsf24F* | TTCCTACACCAACTCCAACA | *PvHsf24R* | CCTGTCCAAACCCCTGC |
| *PvHsf29F* | GTGCCTGCTCCTTTCCTC | *PvHsf29R* | GGCGAACGAAACTTGAGAA |
| *Pvactin11F* | TGCATACGTTGGTGATGAGG | *Pvactin11R* | AGCCTTGGGGTTAAGAGGAG |
